# Fracture resistance of ceramic vonlays fabricated from different CAD/CAM materials restoring premolars after cyclic loading (an in vitro study)

**DOI:** 10.1186/s12903-026-08493-2

**Published:** 2026-05-09

**Authors:** Ahmed Mabrouk AL-Mazzaly, Wegdan Abdel-Fattah, Dina Mohamed Nasr

**Affiliations:** 1 Conservative Dentistry Department, Faculty of Dentistry, Alexandria University, P. O. Box: 21527, Champollion St., Azarita, Alexandria, Egypt; 2 Conservative Dentistry, Faculty of Dentistry, Alexandria University, Alexandria, Egypt

**Keywords:** CAD/CAM ceramics, Lithium disilicate, Zirconia-reinforced ceramics, Polymer-infiltrated ceramic, Fracture resistance

## Abstract

**Introduction:**

The advancement of indirect ceramic restorations helps to establish new boundaries between conservative practices and prosthetics, thus Vonlay restorations were introduced to achieve the desired results in cases that involve the occlusal surface and extends to entire buccal surface due to either esthetic or functional considerations.

**Aim of the study:**

This in vitro study evaluated the fracture resistance and failure modes of ceramic vonlay restorations fabricated from four CAD/CAM materials: lithium disilicate, zirconia-reinforced lithium silicate, super high-translucent zirconia, and polymer-infiltrated ceramic after cyclic loading.

**Materials and methods:**

Fifty-six extracted human maxillary premolars were prepared and restored with vonlays from lithium disilicate glass ceramics (control group), zirconia-reinforced lithium silicate (ZLS), ultra-translucent monolithic zirconia, and polymer-infiltrated ceramic networks (PICNs) (*n* = 14/group). Restorations were milled, finished, and adhesively luted with dual-cure resin cement. All specimens underwent 500,000 cycles of mechanical loading (100 N, 1 Hz) before static fracture testing. Failure modes were assessed microscopically. Data were analyzed using one-way ANOVA, Tukey’s post hoc, and Chi-square/Fisher’s exact tests (α = 0.05).

**Results:**

Ultra-translucent monolithic zirconia showed the highest fracture resistance, followed by lithium disilicate glass ceramics and zirconia-reinforced lithium silicate (ZLS) while polymer-infiltrated ceramic networks (PICNs) showed the lowest values (*p* < 0.001). Failure patterns differed significantly among groups (*p* = 0.005), with polymer-infiltrated ceramic networks (PICNs) having more restorable failures.

**Conclusion:**

Ceramic material significantly influences the mechanical performance of vonlay restorations. High-strength ceramics, such as lithium disilicate glass ceramics, ultra-translucent monolithic zirconia, offer superior resistance and clinical predictability in premolars under cyclic loading.

**Supplementary Information:**

The online version contains supplementary material available at 10.1186/s12903-026-08493-2.

## Introduction

Preservation of natural tooth structure is a cornerstone of modern conservative dentistry. This principle becomes especially critical in cases involving substantial loss of hard tissue, where the structural and functional integrity of the remaining tooth must be maintained to ensure long-term success [1, 2]. Beyond mechanical reinforcement, the biological and esthetic preservation of the dentition plays a pivotal role in treatment planning—particularly as patients increasingly seek minimally invasive solutions with high esthetic outcomes [3–5]. 

Partial-coverage ceramic restorations have gained popularity as conservative alternatives to full-coverage crowns. These include inlays, onlays, and overlays, classified according to their extent of cuspal coverage [5]. A more recent evolution in this category is the vonlay, also referred to as a “veneerlay.” This hybrid restoration combines the occlusal coverage of an onlay or overlay with a buccal veneer extension, allowing for simultaneous restoration of both function and esthetics [6, 7]. Vonlays are particularly suited for posterior teeth—such as maxillary premolars—that require extensive occlusal and facial rehabilitation without compromising sound tooth structure [6–9]. 

Recent advancements in CAD/CAM technology have revolutionized restorative workflows, enabling the efficient fabrication of indirect restorations from a broad range of high-performance ceramics. These include lithium disilicate glass ceramics, zirconia-reinforced lithium silicate (ZLS), ultra-translucent monolithic zirconia, and polymer-infiltrated ceramic networks (PICNs). Each material differs in its mechanical behavior, optical properties, and clinical indications, making material selection a key factor in restoration longevity [10–14]. 

Among these, lithium disilicate stands out as a reinforced glass ceramic containing a dense crystalline network of lithium disilicate crystals within a glass matrix. This structure imparts high mechanical strength along with excellent esthetic characteristics, making it the preferred material for anterior and posterior veneers, inlays, onlays, crowns, and partial-coverage restorations [15–17]. Recent advancements in lithium disilicate formulations have enabled clinicians to achieve durable restorations for posterior teeth using conservative designs, with minimal material thickness—sometimes as little as 1.0 mm—without compromising strength or performance [15, 18, 19]. 

To enhance the mechanical performance of glass ceramics, researchers have modified their composition by incorporating approximately 10% zirconia particles by weight. This innovation led to the development of zirconia-reinforced lithium silicate (ZLS), a material that combines the favorable attributes of both lithium silicate and zirconia ceramics—namely, high flexural strength and superior esthetics [19–21]. 

Zirconia-based ceramics have also garnered significant attention in restorative dentistry due to their excellent mechanical properties and biocompatibility. Traditionally, zirconia cores have been veneered with more translucent glass ceramics to improve esthetics. However, veneered restorations are prone to complications such as chipping and delamination of the outer ceramic layer. In response, full-contour monolithic zirconia restorations have been introduced, offering sufficient esthetic quality and eliminating the need for a veneering layer, thereby improving structural integrity and reducing failure rates [22–24]. 

Efforts to improve the optical properties of zirconia have resulted in material modifications aimed at increasing translucency. This has been achieved through the adjustment of the yttrium oxide (Y₂O₃) content—typically raising it from 3 mol% to 5 mol% or more—as well as by minimizing porosity through the reduction or elimination of alumina-based sintering additives. These changes promote a greater proportion of the cubic zirconia phase, which exhibits enhanced translucency compared to the tetragonal phase [25]. 

As a result of these developments, high-translucency fully cubic zirconia has been increasingly used in partial-coverage restorations [26, 27]. In vitro studies have demonstrated that monolithic zirconia exhibited greater fracture resistance than both veneered zirconia restorations and lithium disilicate ceramics, making it a viable option for high-stress areas in the posterior dentition [28]. 

Another promising material for indirect restorations is the polymer-infiltrated ceramic network (PICN). This hybrid material features a dominant ceramic matrix reinforced by an infiltrated polymer network. PICN offers favorable mechanical behavior, particularly for posterior restorations, due to its ability to absorb and dissipate occlusal forces effectively. Additionally, it exhibits reduced brittleness compared to conventional glass ceramics, which may contribute to its clinical longevity [29]. CAD/CAM polymer restorative materials have been continually improved to be used as an alternative to glass-ceramics [30, 31]. 

Currently, there is limited evidence guiding the selection of the most suitable material for vonlay restorations, particularly concerning how these materials perform under cyclic loading conditions. This gap in knowledge is significant, as cyclic fatigue may influence the longevity and clinical success of such restorations [32, 33]. 

As a result, the current in vitro study aims to investigate the effect of cyclic loading on the fracture resistance of vonlay restorations made from four different CAD/CAM materials: lithium disilicate ceramic, zirconia-reinforced lithium silicate ceramic, ultra-translucent monolithic zirconia, and polymer-infiltrated ceramic network material.

The null hypothesis was that there would be no significant difference in fracture resistance among the tested materials after exposure to simulated functional loading.

## Materials and methods

The Faculty of Dentistry Institutional Review Board of Alexandria University (IRB No. 001056 - IORG 0008839) gave its approval for this in vitro study. All procedures were carried out in conformity with the Declaration of Helsinki and the ethical standards established by the Faculty of Dentistry’s Research Ethics Committee.

### Study design “clinical trial number: not applicable.”

In vitro experimental study evaluates and compares the fracture resistance of premolars restored with vonlay restorations fabricated from different CAD/CAM materials. A total of fifty-six (*N* = 56) sound human maxillary premolars were selected and equally assigned into four groups (*n* = 14 per group), based on the type of restorative material used.

An overview of the materials used in the present study is listed in Table 1.

**Table 1:** Composition of materials used in the study

Material	Classification	Composition	Manufacturer
IPS e-max block	CAD/CAM lithium disilicate ceramic	57–80% SiO _2_, 11–19% Li_2_O, 0–13% K_2_O, 0–11% P_2_O_5_, 0–8% ZrO_2_, 0–8% ZnO_2_, Coloring oxides	IPS e.max CAD Ivoclar vivadent / Schaan - Liechtenstein
Vita Suprinity block	CAD/CAM Zirconia reinforced lithium silicate ceramic	Lithium silicate with 10% ZrO_2_	Vita Suprinity / Vita Zahnfabrik / Bad Sackingen – Germany
Ceramill Zolid FX block	CAD/CAM Super High Translucent Zirconia	Less than 99% ZRO_2_, HFO_2_, Y_2_O_3_, 8.5–9.5% Y_2_O_3_ lwss than 0.5% AL_2_O_3_ and other oxides	Zolid FX block (Ceramill Zi; Amann Girrbach, Koblach, Austria
Vita Enamic Block	CAD/CAM Polymer-infiltrated ceramic	**Ceramic Component**: 58–63%,SiO_2_, 20–23%, Al_2_O_3_, 6–11% Na_2_O, 4–6% K_2_O, 0.5–2% B_2_O_3_, < 1% CaO, < 1% TiO_2_ **Polymer Component** : Methyl methacrylate (MMA) free surface modified polymethylmethacrylate(PMMA)	Vita Enamic / Vita Zahnfabrik / Bad Sackingen – Germany
Adhesive resin cement:			
1- Duo-Link Universal	Adhesive Resin Cement	**Base**: Bis-GMA, triethyleneglycol dimethacrylate, urethane dimethacrylate, glass filler **Catalys**t: Bis-GMA, triethyleneglycol dimethacrylate, glass filler	BISCO, USA
2-Universal Primer	Universal Primer	Ytterbium Fluoride, Urethane Dimethacrylate, Ytterbium Oxide-Silica, Tetrahydrofurfuryl Methacrylate,, Bisphenol A Diglycidylmethacrylate, Trimethylolpropane trimethacrylate	BISCO, USA
3- Phosphoric Acid etchant 37%	Etching Agent	37% Phosphoric Acid andBenzalkonium Chloride BAC	BISCO, USA
4-Porcelain etchant	Ceramic Etching	9% Hydrofloric Acid, water, thickener, surfactant and dye	BISCO, USA
5-Porcelain Primer	Silane coupling agent	1% aminosilano, 70–80% ethanol and 20–30% water	BISCO, USA
6- Z prime Plus	Zirconia conditioner	MDP, a phosphate monomer, and BPDM, a carboxylate monomeand silane monomer	BISCO, USA

### Sample size calculation

The sample size was estimated assuming a 5% alpha error and 80% study power. The mean (SD) fracture resistance was 582.3 (119.6) and 704.3 (139.3) for lithium disilicate and Zirconia reinforced lithium silicate, respectively [19], and it was 791.53 (80.52) for polymer infiltrated ceramic [29] and 1843.66 (306.20) for High Translucent Zirconia [27]. The highest sample size was calculated based on comparisons between the lithium disilicate, Zirconia reinforced lithium silicate, and polymer infiltrated ceramic using F test and pooled SD = 161.41. The sample size was estimated to be 13 sample per group, this was increased to 14 sample to make up for processing errors. Total sample = number per group x number of groups = 14 × 4 = 56 samples. The sample size was based on Rosner’s method calculated by G*Power 3.1.9.7.

### Specimen selection and preparation

Fifty-six freshly extracted maxillary premolars, free from caries, restorations, cracks, and structural anomalies, were selected. Teeth were extracted for orthodontic or periodontal reasons and disinfected in chloramine-T solution for one week. After ultrasonic cleaning, they were stored in distilled water until use. Standardization of tooth selection was carefully performed. Extracted maxillary premolars with comparable dimensions were selected. Mesiodistal and buccopalatal dimensions were measured using a manual caliper, and only teeth within a narrow range of variation (mesiodistal: 6.5–7.5 mm; buccopalatal: 8.5–9.5 mm) were included to minimize variability in mechanical performance [34]. 

To simulate the periodontal ligament, a 0.2–0.3 mm layer of utility wax was applied to the root surface, then replaced with light-body polyvinyl siloxane. Specimens were embedded in self-curing acrylic resin using a custom copper mold, with 2 mm of the root exposed apical to the cementoenamel junction (CEJ) for standardization. Silicone putty indices were fabricated before preparation to guide standardized tooth reduction [9]. 

### Tooth preparation for vonlay restorations

**Fig. 1:**
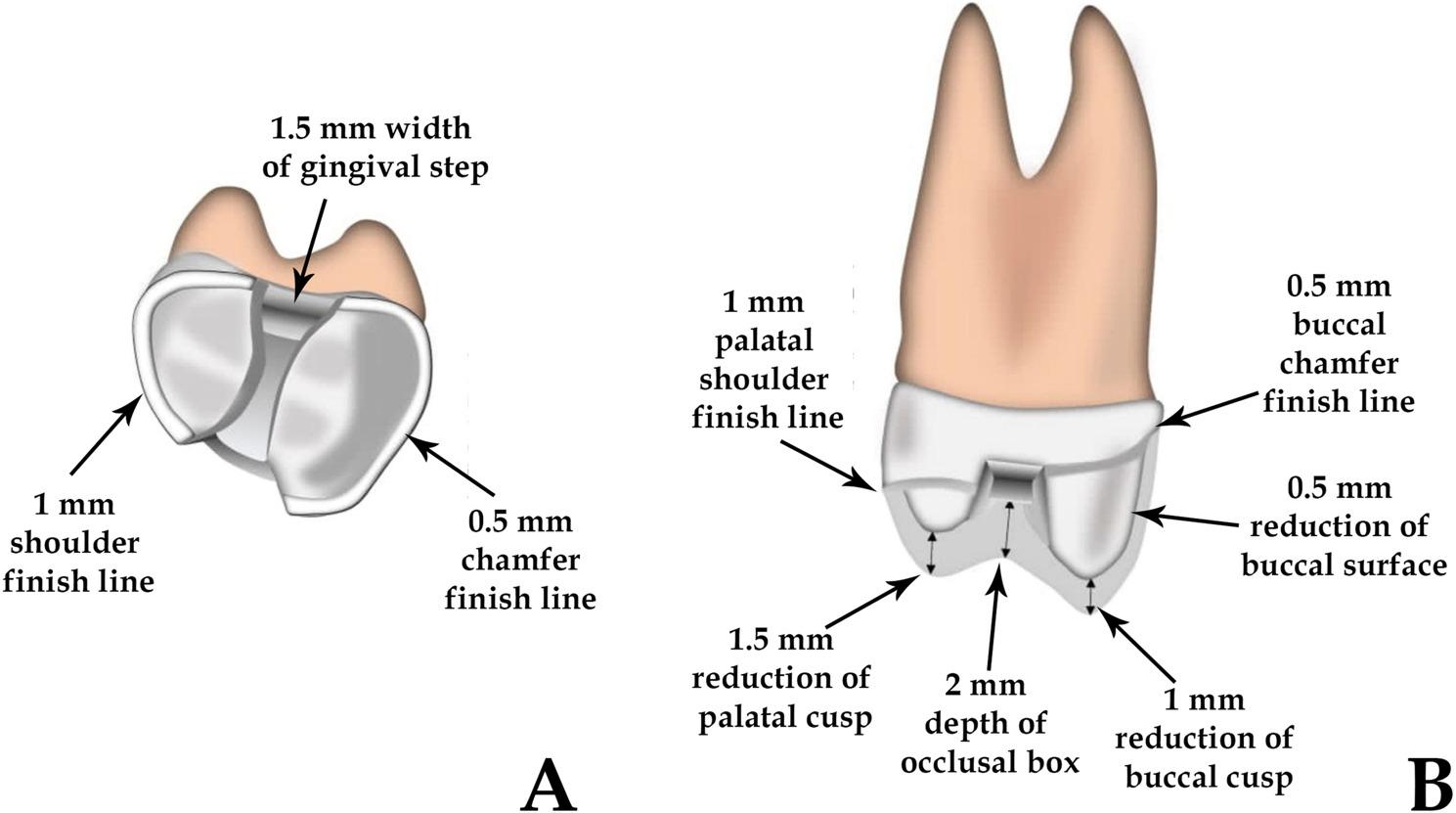
Illustrations showing, Vonlay preparation design (**a**) occlusal view, and (**b**) proximal view

All teeth were prepared to receive Vonlay restorations as presented in (Fig. 1). Each tooth was individually prepared to better simulate real clinical conditions and anatomical variability encountered in routine practice. Although this may introduce slight operator-induced variation, standardization protocols using silicone indices and calibrated preparation guides were employed to minimize inconsistencies. All teeth were prepared to receive vonlay restorations, following a standardized MOD inlay design with an extended buccal veneer component. The cavity depth was set to 2 mm occlusally; buccal and palatal walls had a 12° divergence. The isthmus width was one-third of the intercuspal distance [9]. 

**Fig. 2:**
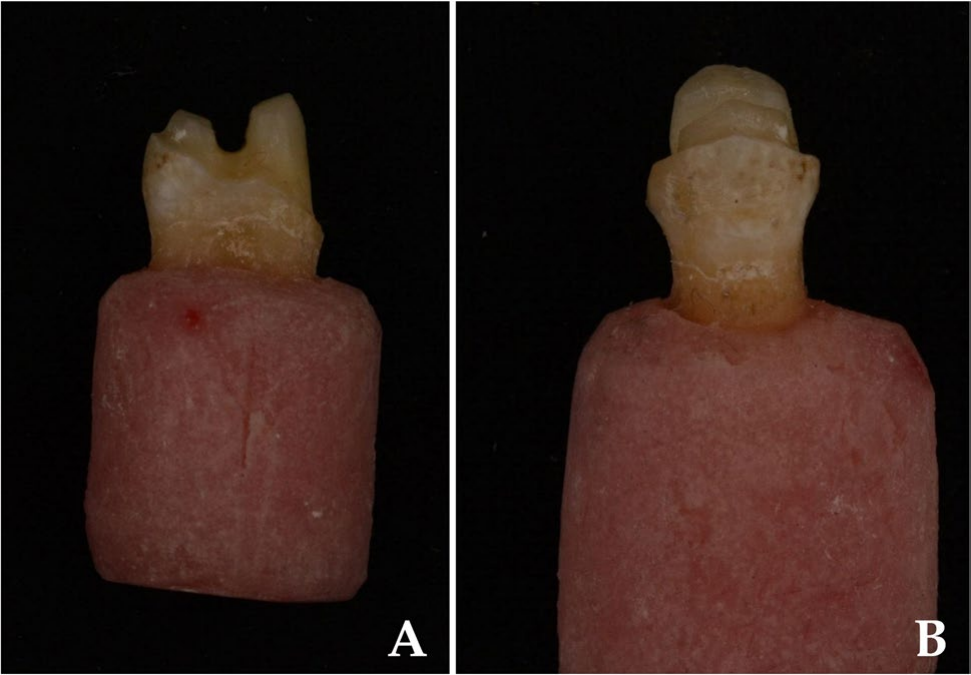
Prepared maxillary premolar to receive vonlay restorations (**a**) proximal view, and (**b**) palatal view

Cusp reduction included 1.0 mm on the buccal and 1.5 mm on the palatal side. A 0.5 mm chamfer finish line was placed on the buccal surface approximately 1 mm above the cervical margin, while a 1 mm shoulder finish line was prepared on the palatal surface. All internal angles were rounded and margins smoothed to minimize stress concentration [9, 19, 32] (Fig. 2).

To ensure consistency in tooth preparation, a standardized preparation protocol was followed. The same type of diamond burs was used throughout the study, and burs were replaced after every 5 preparations to ensure consistent cutting performance and minimize variability, in line with the need for standardization reported in the literature [35]. 

### Group allocation and materials

The prepared Specimens were randomly assigned into four groups (*n* = 14 per group) based on the CAD/CAM material used:

- Group 1: Lithium disilicate (IPS e.max CAD, Ivoclar Vivadent, Liechtenstein) (control group)
- Group 2: Zirconia-reinforced lithium silicate (VITA Suprinity, Vita Zahnfabrik, Germany)
- Group 3: Ultra-translucent monolithic zirconia (Ceramill Zolid FX, Amann Girrbach, Austria)
- Group 4: Polymer-infiltrated ceramic network (VITA Enamic, Vita Zahnfabrik, Germany)

The composition and classification of the materials are detailed in Table 1.

### CAD/CAM fabrication of vonlay restorations

**Fig. 3:**
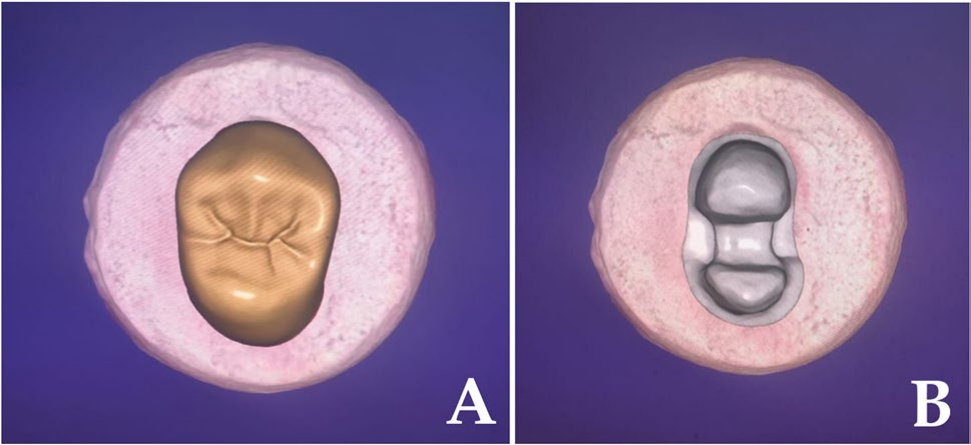
Representative images of the designed vonlay restoration (**a**). the underlying scanned tooth preparation design (**b**)

Teeth were scanned using an intraoral scanner (Medit i700, Medit Corp., South Korea), and restorations were designed using CAD software (exocad GmbH, Germany), maintaining standardized anatomical thickness and a 50 μm cement space (Fig. 3).

**Fig. 4:**
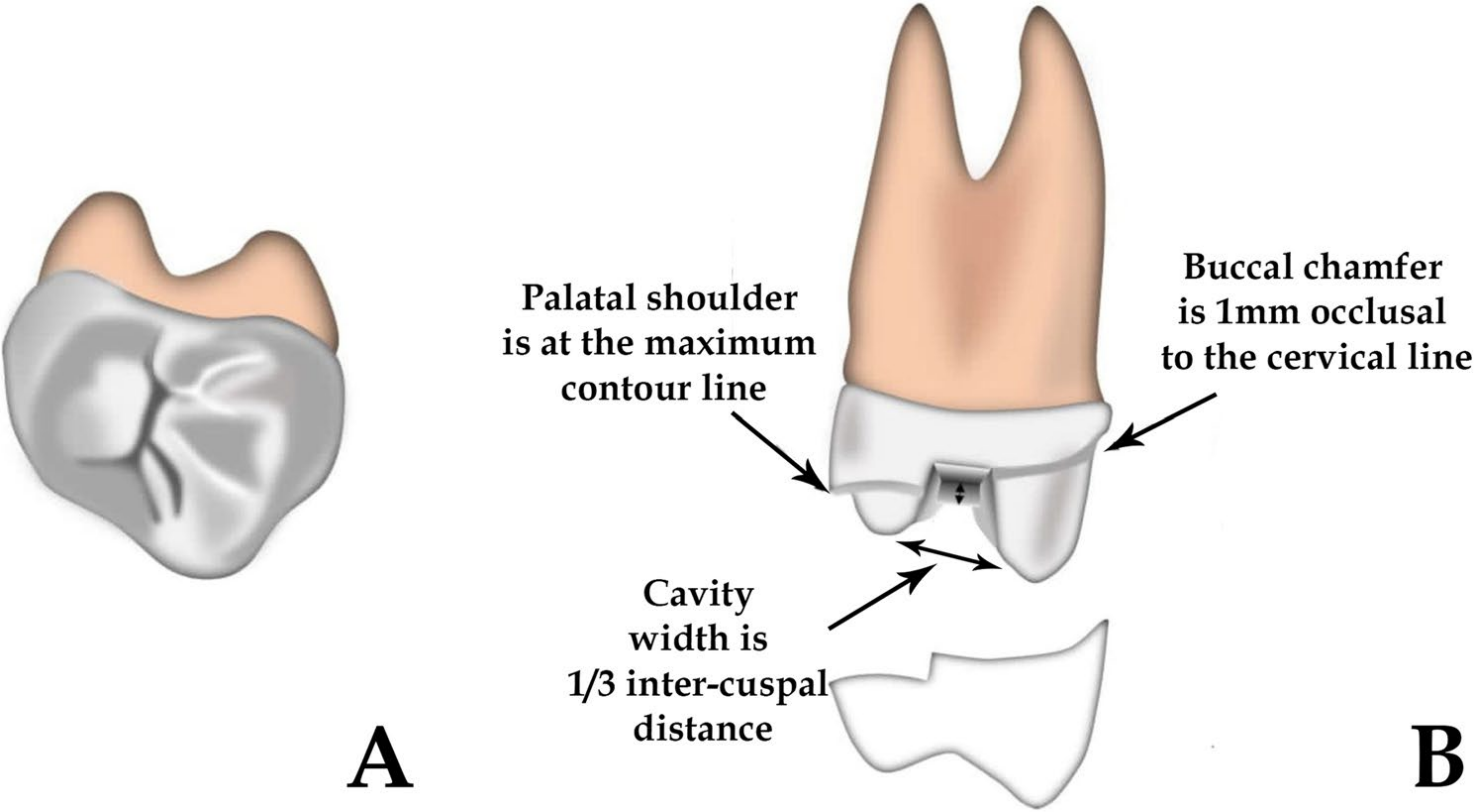
Illustrations showing, Vonlay restoration design (**a**) occlusal view (**b**) proximal view

Restorations were milled using a CAM milling unit (KAVO Everest Engine). Lithium disilicate glass ceramics and zirconia-reinforced lithium silicate (ZLS) were crystallized post-milling according to manufacturer protocols. Ultra-translucent monolithic zirconia was sintered at 1450 °C for 2 h. Polymer-infiltrated ceramic networks (PICNs) restorations were used directly post-milling without additional treatment. All restorations were finished and glazed per manufacturer recommendations [36] (Figs. 4).

### Surface treatment and bonding procedure

#### Surface treatment for CAD/CAM restorations [37–39] 

Hydrofluoric acid etching 9.5% (Hydrofluoric acid etching 9.5%, BISCO USA) was applied to the fitting surface of lithium disilicate Vonlay, Zirconia reinforced lithium silicate Vonlay and Polymer-infiltrated Vonlay for 20 s then was rinsed with forceful water spray, followed by rinsing for 20 s under running water, then they was dried with oil free air spray for 30 s. This was followed by application of a single layer of silane coupling agent to the fitting surface using fine brushes and it was allowed to react for 60 s then air dried with oil free air spray.

Zirconia surface pretreatment was done by airborne particle abrasion with alumina particles to the intaglio fitting surface of zirconia. The vonlay restorations were air-abraded using 50 μm Aluminum Oxide particles (Al_2_O_3_) from a distance of 10 mm for 20 s at 2.8 bar pressure, followed by application of zirconia primer Z-Prime Plus (combination of two active monomers, MDP, a phosphate monomer, and BPDM, a carboxylate monomer, BISCO, USA). It was applied for 60 s then was dried be air syringe [37–39]. 

#### Surface treatment for prepared teeth

- Surface treatment of the prepared teeth were done using (37% phosphoric acid etching gel, BISCO, USA) for 15 s, then rinsed thoroughly with a stream of water for at least 15 s and dried with compressed air just for cleaning.
- Universal primer (Universal primer, BISCO, USA) was applied by micro-brushes on the entire prepared cavity surfaces. Two separate coats were applied, then scrubbing the preparation with a microbrush for 10–15 s per coat without light curing between coats. Excess solvent was evaporated by strongly air-drying with an air syringe for at least 10 s; there should be no visible movement of the adhesive.

#### Cementation procedures

**Fig. 5:**
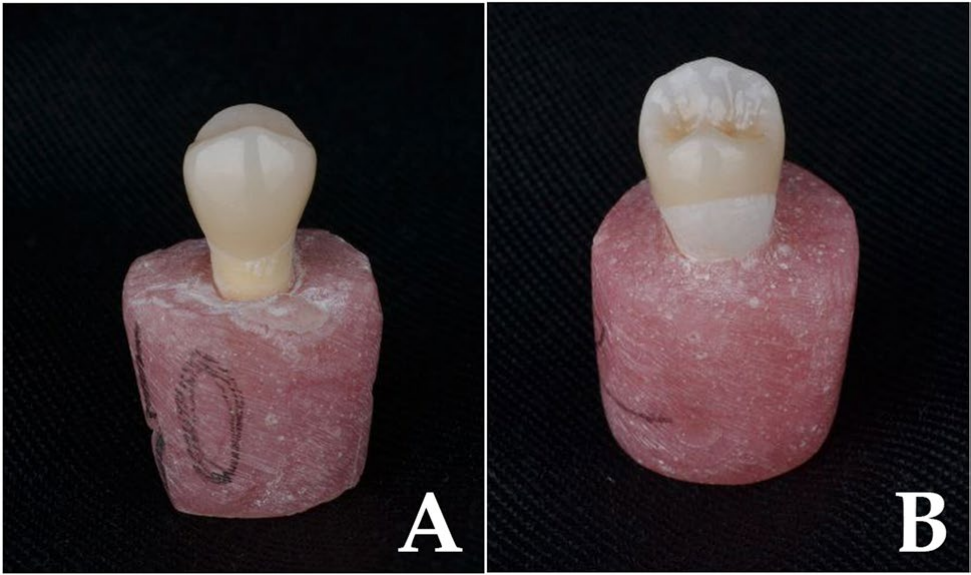
Cemented vonlay (**a**) buccal view (**b**) palatal view

Dual cure self-adhesive resin cement was used to cement the Vonlays. Cement material was dispensed inq2e43to the intaglio surface of each Vonlay and prepared teeth. The bonded Vonlay restorations to the teeth were placed under static load 1Kg weight to have a uniform thickness of resin cement and they were spot-cured for 2 s from each direction to remove all excess resin cement. Followed by light-curing for 40 s using LED visible light with light intensity of 1200 mW/cm2, for each quarter surface (mesio-facial, disto-facial, disto-lingual, mesio-lingual) according to the manufacturer’s instructions. Static load was left in place till 3 min to allow for the setting time of Dual cure adhesive resin cement [37–39] (Fig. 5).

### Cyclic loading of the restored teeth

As the number of chewing cycles per day was estimated at approximately 800–1400, a moderate 500,000 cycles are considered as the functioning cycle for the restored teeth per year. Also, physiologic occlusal forces in the human mouth show variability between individuals and range between 10 and 120 N during mastication and swallowing [33, 40]. Thus, all restored teeth with Vonlay restorations were submitted to mechanical cyclic loading with vertical load of 100 N for 500,000 cycles and 1 Hz frequency. The mechanical cyclic loading was applied to the center of the occlusal surface in contact with both cusp ridges with a stainless-steel antagonist with a rounded end that is 4 mm in diameter [41]. 

### Fracture resistance testing

**Fig. 6:**
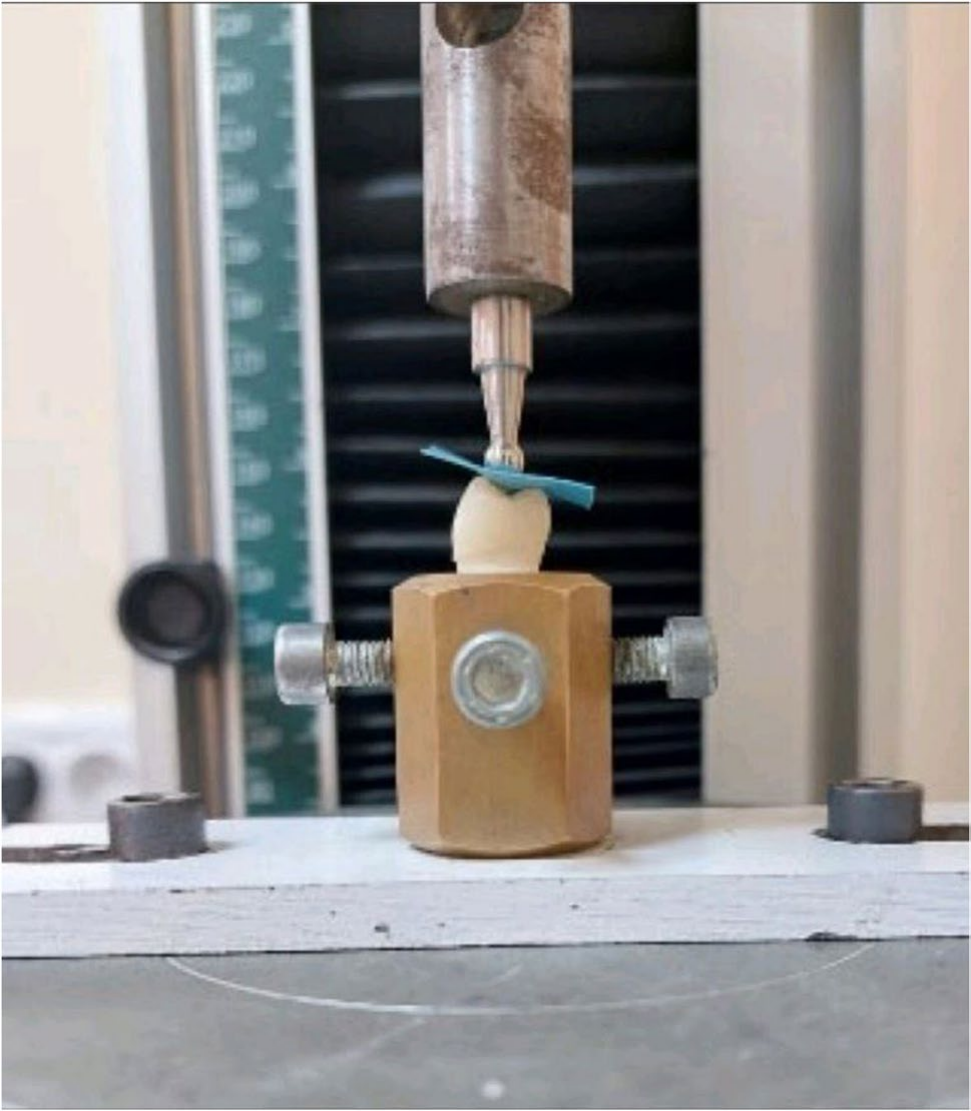
Fracture resistance test. (Tinius Olsen Model 5ST, Tinius Olsen Ltd., England, 2018)

The Vonlay restorations that survived cyclic load were then loaded to failure in compression in a universal testing machine (Tinius Olsen Model 5ST, Tinius Olsen Ltd., England, 2018) was used to evaluate the fracture resistance of the restored teeth. The force was applied perpendicular the long axis of each restored teeth. The restored teeth were attached to the lower arm of the machine, while the 8 mm stainless steel ball was fixed on the upper arm of the machine. Compressive force was applied with a speed of (0.5 mm/min), and the forces were shared by the triangular ridges of the cusps. A 0.6 mm rubber sheet was placed between ball end and restored teeth for homogenous distribution of the load applied. Each restored tooth was loaded until fracture occurred and the forces in Newton were recorded and tabulated [36, 42] (Fig. 6).

### Mode of failure

After fracture resistance test, all the fractured surfaces of Vonlay restorations were carefully examined at 20x magnification to identify the failure mode using Stereomicroscope and they were classified into four types according to Guess (2013): [33].

- Mode I: Extensive crack formation within the ceramic.
- Mode II: Cohesive fracture within the ceramic.
- Mode III: Fracture within the ceramic and tooth structures.
- Mode IV: Longitudinal ceramic and tooth fracture involving the root.

### Statistical analysis

Data normality was verified using the Shapiro-Wilk test and Q-Q plots. One-way ANOVA with Tukey’s post hoc test was used for comparing fracture resistance. Chi-square and Fisher’s exact tests assessed associations between materials and failure types. Significance was set at *p* < 0.05. Statistical analysis was conducted using SPSS (IBM Corp., NY, USA).

## Results

### Fracture resistance

**Fig. 7:**
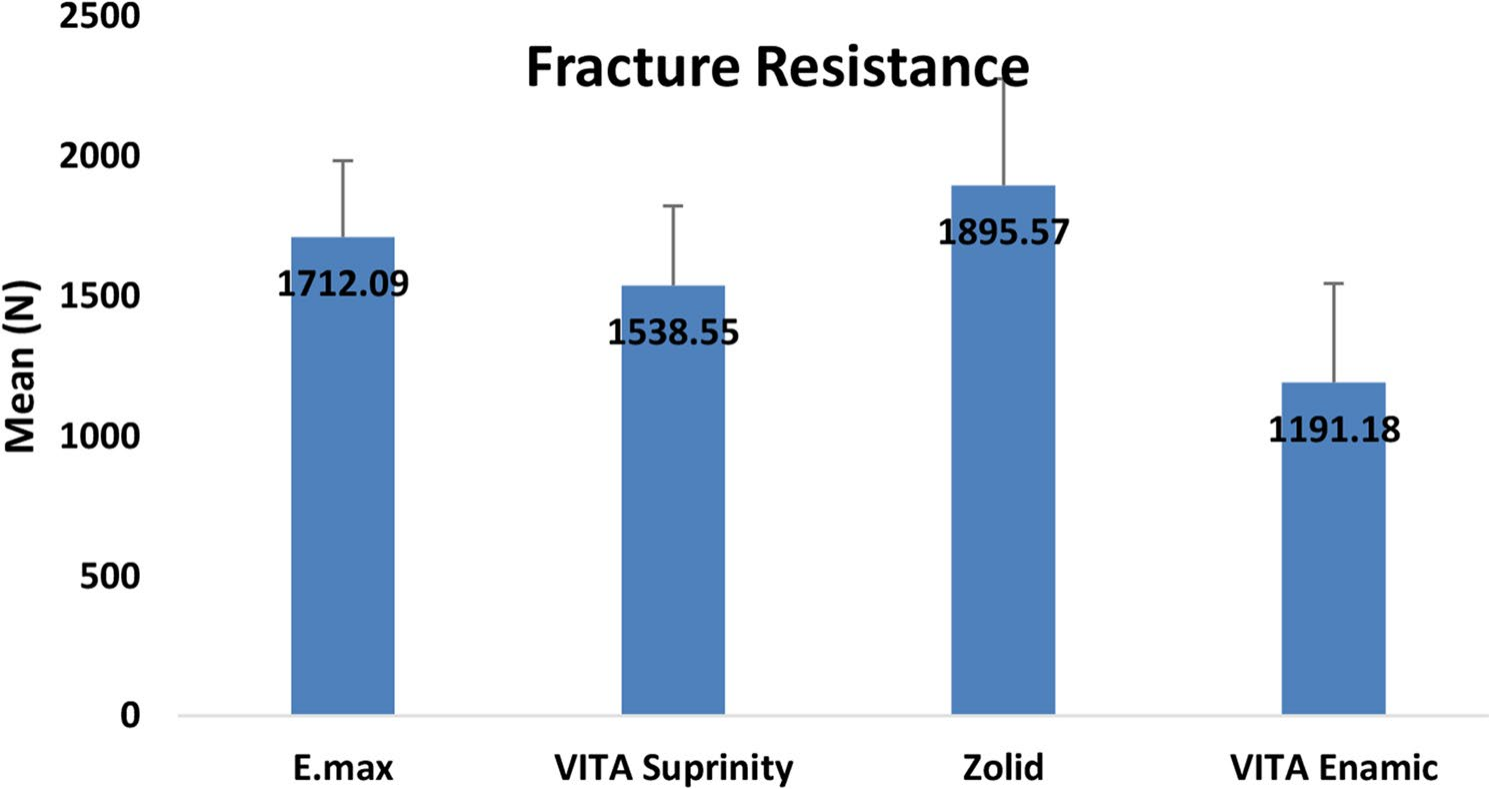
Chart showing a comparison of fracture resistance between four studied groups

The mean fracture resistance values (± SD) for the four material groups are summarized in Table 2. Ultra-translucent monolithic zirconia (Ceramill Zolid FX) (Group 3) exhibited the highest mean fracture resistance (1895.57 ± 381.69 N), followed by lithium disilicate glass ceramics (IPS e.max CAD) (Group 1) (Control Group) (1712.09 ± 273.47 N), zirconia-reinforced lithium silicate (ZLS) (VITA Suprinity) (Group 2) (1538.55 ± 285.25 N), and polymer-infiltrated ceramic networks (PICNs) (VITA Enamic) (Group 4) (1191.18 ± 355.57 N), which recorded the lowest values (Table 2) (Fig. 7).

**Table 2:** Comparison of fracture resistance among the study groups

	E.max (*n* = 14)	VITA Suprinity (*n* = 14)	Zolid (*n* = 14)	VITA Enamic (*n* = 14)
Mean ± SD	1712.09 ± 273.47	1538.55 ± 285.25	1895.57 ± 381.69	1191.18 ± 355.57
Median	1667.83	1594.15	1803.47	1309.38
Min – Max	1357.12–2526.64	1007.39–1898.25	1511.20–2911.61	502.79–1721.07
F test (*p* value)	11.76 (< 0.001*)			

*Statistically difference at *p* value < 0.05

One-way ANOVA revealed a statistically significant difference among the groups (F = 11.76, *p* < 0.001). Tukey’s post hoc pairwise comparisons showed that:

Polymer-infiltrated ceramic networks (PICNs) (VITA Enamic) had significantly lower fracture resistance than ultra-translucent monolithic zirconia (Zolid FX) (*p* < 0.001), lithium disilicate glass ceramics (IPS e.max CAD) (*p* = 0.001), and zirconia-reinforced lithium silicate (ZLS) (VITA Suprinity) (*p* = 0.034). Ultra-translucent monolithic zirconia (Zolid FX) exhibited significantly higher values than zirconia-reinforced lithium silicate (ZLS) (VITA Suprinity) (*p* = 0.028). No statistically significant difference was found between lithium disilicate glass ceramics (IPS e.max CAD) and zirconia-reinforced lithium silicate (ZLS) (VITA Suprinity) (*p* = 0.503), or between lithium disilicate glass ceramics (IPS e.max CAD) and ultra-translucent monolithic zirconia (Zolid FX) (*p* = 0.455) (Table 3).

**Table 3:** Pairwise comparison regarding fracture resistance between the study groups

Groups	Compared to	Mean difference	95% CI	*p* value
E.max	VITA Suprinity	173.54	-154.71, 501.78	0.503
	Zolid	-183.48	-511.73, 144.76	0.455
	VITA Enamic	520.91	192.66, 849.15	0.001*
VITA Suprinity	Zolid	-357.02	-685.26, -28.78	0.028*
	VITA Enamic	347.37	19.13, 675.61	0.034*
Zolid	VITA Enamic	704.39	376.15, 1032.64	< 0.001*

*Statistically significant difference at *p* value < 0.05

### Mode of failure

**Fig. 8:**
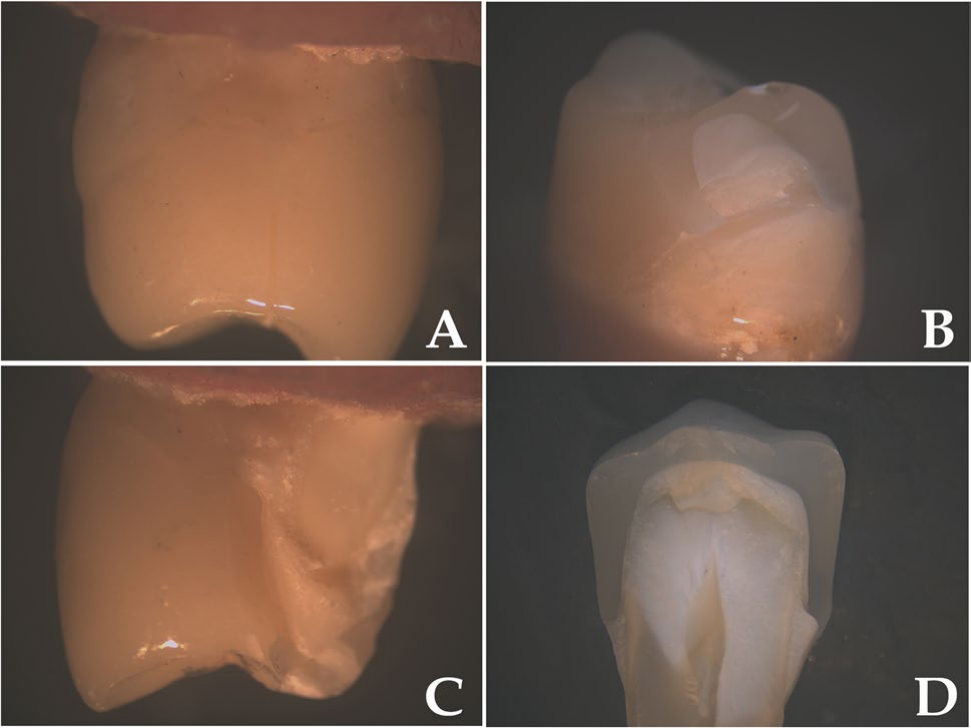
Illustrate the fracture mode observed in this study; as (**a**) Mode I: Extensive crack formation within the ceramic. **b** Mode II: Cohesive fracture within the ceramic. **c** Mode III: Fracture within the ceramic and tooth structures. **d** Mode IV: Longitudinal ceramic and tooth fracture involving the root

**Fig. 9:**
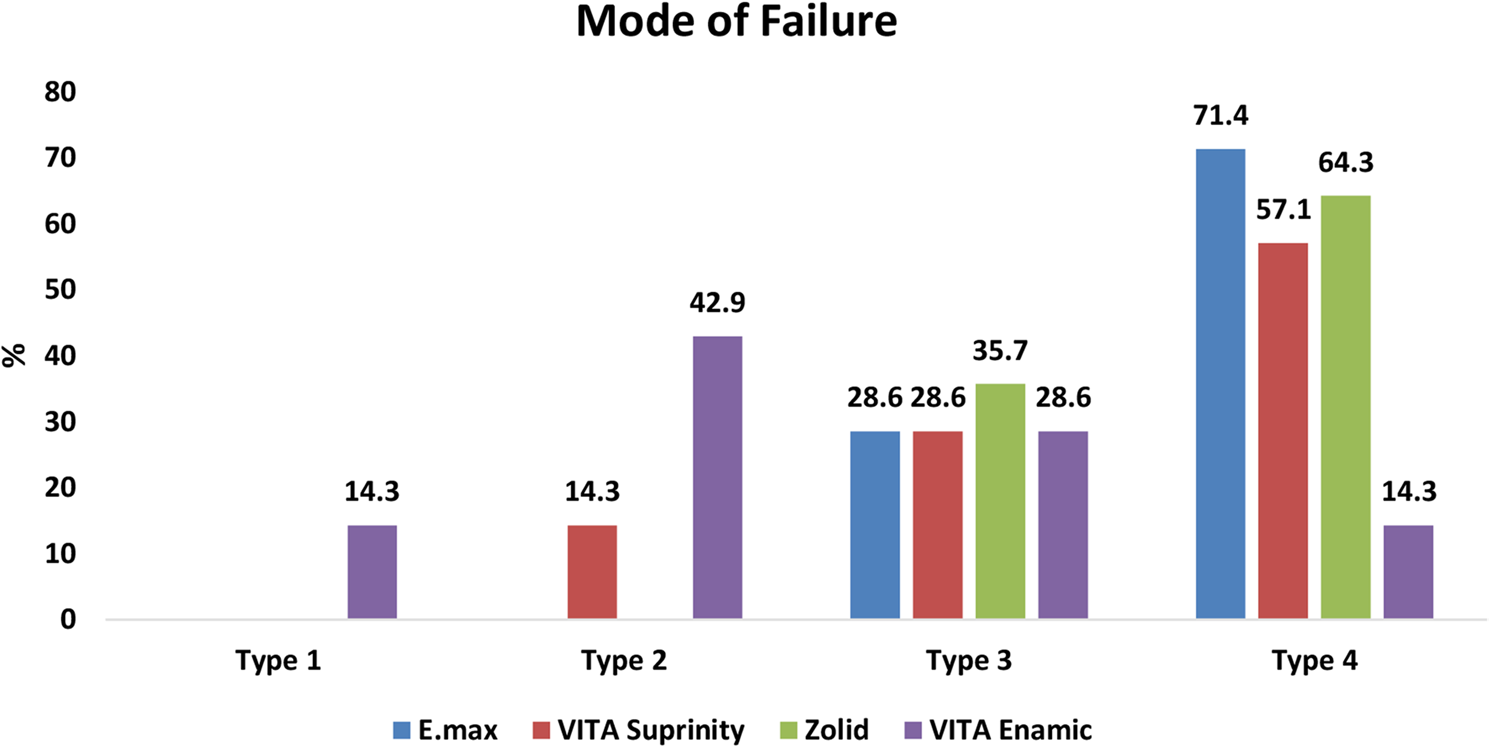
Chart showing a comparison of mode of failure between four studied groups

The distribution of failure modes differed significantly among the four groups (χ² = 23.52, *p* = 0.005) (Table 4) (Figs. 8 and 9).

**Table 4:** Comparison of mode of failure between the study groups

	E.max (*n* = 14)	VITA Suprinity (*n* = 14)	Zolid (*n* = 14)	VITA Enamic (*n* = 14)
	*n* (%)			
Type 1	0 (0%)	0 (0%)	0 (0%)	2 (14.3%)
Type 2	0 (0%)	2 (14.3%)	0 (0%)	6 (42.9%)
Type 3	4 (28.6%)	4 (28.6%)	5 (35.7%)	4 (28.6%)
Type 4	10 (71.4%)	8 (57.1%)	9 (64.3%)	2 (14.3%)
X^2^ test (*p* value)	23.52 (0.005*)			

*Statistically significant difference at *p* value < 0.05

Polymer-infiltrated ceramic networks (PICNs) (VITA Enamic) showed the highest incidence of Mode II failures (cohesive within ceramic) at 42.9% and was the only group to exhibit Mode I (non-separated internal cracks) failures (14.3%). Mode IV (catastrophic root-involving fracture) was most frequent in ultra-translucent monolithic zirconia (Zolid FX) (64.3%), lithium disilicate glass ceramics (IPS e.max CAD) (71.4%), and zirconia-reinforced lithium silicate (ZLS) (VITA Suprinity) (57.1%). Mode III (combined tooth and ceramic fracture) occurred relatively equally across all groups (Table 4).

Pairwise comparisons revealed significant differences in failure patterns between:

Polymer-infiltrated ceramic networks (PICNs) (VITA Enamic) and lithium disilicate glass ceramics (IPS e.max CAD) (*p* = 0.004) and with Ultra-translucent monolithic zirconia (Zolid FX) (*p* = 0.006). No significant differences were noted between lithium disilicate glass ceramics (IPS e.max CAD) and ultra-translucent monolithic zirconia (Zolid FX) (*p* = 1.000) or between zirconia-reinforced lithium silicate (ZLS) (VITA Suprinity) and the other materials (Table 5).

**Table 5:** Pairwise comparison regarding mode of failure between the study groups

Groups	Compared to	*p* value
E.max	VITA Suprinity	0.329
	Zolid	1.00
	VITA Enamic	0.004*
VITA Suprinity	Zolid	0.338
	VITA Enamic	0.055
Zolid	VITA Enamic	0.006*

*Statistically significant difference at *p* value < 0.05

## Discussion

In this in vitro study, all vonlay restorations were adhesively cemented using a dual-cure universal resin cement, applied following a consistent protocol across all groups. The use of two coats of adhesive, as recommended by the manufacturer, likely contributed to uniform bond strength by enhancing monomer penetration and surface coverage, which are critical to the clinical longevity of bonded ceramic restorations [37, 39]. 

A potential limitation of this study is the use of different surface treatment protocols according to the material type. While these protocols reflect clinically recommended procedures, they may introduce a confounding factor, as the observed fracture resistance values may be influenced not only by the intrinsic properties of the materials but also by differences in the material–adhesive interface. Therefore, the results should be interpreted with consideration of this limitation [43]. 

The integration of CAD/CAM technology has significantly enhanced the precision and predictability of indirect restorations, reducing chairside time while maintaining high esthetic and functional standards [44]. The vonlay design—combining an onlay with a buccal veneer—offers a conservative and highly esthetic alternative to full-coverage crowns, particularly in posterior regions where structural preservation and esthetic outcome are equally important. As supported by Guess et al. (2013), Kim et al. (2017) and Al-Akhali et al. (2019), vonlays are ideally suited for structurally compromised maxillary premolars located within the esthetic zone [33, 45, 46]. 

The null hypothesis of the present study was rejected, as statistically significant differences were observed in fracture resistance among the tested CAD/CAM materials. Ceramill Zolid FX demonstrated the highest fracture resistance, followed by IPS e.max CAD and VITA Suprinity, while VITA Enamic exhibited the lowest values. These findings highlight the substantial influence of ceramic composition and microstructure on the mechanical performance of vonlay restorations. Although the tested materials demonstrated fracture resistance values exceeding physiological occlusal forces, these findings should be interpreted with caution, as in vitro results do not necessarily reflect long-term clinical performance.

The present study adopted a material-based reference approach, in which lithium disilicate (IPS e.max CAD) was selected due to its well-established clinical performance and widespread use. This approach enabled a standardized comparison of different CAD/CAM materials within the same minimally invasive vonlay design, reducing variability related to preparation geometry and allowing a clearer evaluation of material-related effects [47, 48]. 

Zolid FX, a super-high-translucent zirconia, achieved the highest resistance, which can be attributed to its dense cubic-phase microstructure. Although the transformation toughening mechanism is reduced due to the high yttria content, its flexural strength remains superior compared to silicate ceramics [40, 49, 50]. These results align with previous studies that confirmed the excellent mechanical stability of monolithic zirconia under cyclic fatigue. Our results partially align with previous studies by Sulaiman et al. (2015) and Al-Zordk et al. (2021), who reported that monolithic zirconia and lithium disilicate ceramics generally exhibit higher fracture strength than resin-ceramic hybrids due to their superior mechanical properties and higher elastic modulus [51, 52]. 

IPS e.max CAD, a lithium disilicate glass ceramic, remains a benchmark material due to its reliable balance between strength and esthetics. Its microstructure—comprising elongated lithium disilicate crystals within a glass matrix—provides crack deflection and resistance to propagation. In the current study, its performance was comparable to VITA Suprinity and slightly inferior to Zolid FX, consistent with the findings of Zimmermann et al. (2017), who reported superior fracture resistance for E.max compared to other silicate-based ceramics [53]. This result in the present study is supported by Coldea et al. (2013) and Elsaka (2016), who highlighted its ability to resist crack propagation under both static and cyclic loading conditions [21, 54]. 

Despite the zirconia reinforcement in VITA Suprinity, its fracture resistance did not significantly exceed that of IPS e.max CAD. This result supports the conclusion of Apel et al. (2007), who proposed that zirconia addition may impair optimal crystal formation and increase matrix viscosity, thereby reducing mechanical gains [55]. Other studies, including Hikita et al. (2007) and Yildiz et al. (2013), reported similar findings, questioning the mechanical advantage of zirconia-reinforced lithium silicate ceramics [56, 57]. However, contrasting evidence from Elsaka and Elnaghy (2016), and Preis et al. (2015), suggested that zirconia addition could improve strength under optimal cementation and in crown applications [21, 58]. Such variability underscores the influence of multiple factors, including restoration geometry, material thickness, and fabrication methods [59]. 

Interestingly, recent findings by Abdelaal et al. (2024) and Fayed et al. (2025) indicated that zirconia-reinforced ceramics may outperform lithium disilicate in fracture resistance for posterior applications [60, 61]. This inconsistency among studies suggests that clinical performance is highly case-dependent and cannot rely solely on material classification.

Among all tested materials, VITA Enamic—a polymer-infiltrated ceramic—exhibited the lowest fracture resistance. Its dual-network structure is designed to absorb occlusal forces and reduce brittleness; however, the inherent mechanical limitations of its polymer phase may compromise strength under fatigue loading. Microcracks at the ceramic-polymer interface, potentially generated during CAD/CAM milling, may act as stress concentrators and promote early failure. These observations are in agreement with Andrade et al. (2018) and Petrini et al. (2013), who noted the susceptibility of hybrid ceramics to mechanical degradation under cyclic stress [62, 63]. Similarly, Al-Akhali et al. (2017) reported inferior performance of PICN materials in high-stress occlusal veneer applications [30]. 

Failure mode analysis revealed important differences between the materials. High-strength ceramics (Zolid FX, IPS e.max CAD, and VITA Suprinity) predominantly exhibited Type IV failures—longitudinal fractures involving both ceramic and tooth structure—indicating catastrophic, non-repairable outcomes. This reflects the brittle nature and high modulus of these ceramics, which can concentrate stress at the adhesive interface under load.

Mode IV failure, characterized as catastrophic root-involving fracture, was the most prevalent failure mode among all tested groups—particularly with lithium disilicate (71.4%), ultra-translucent zirconia (64.3%), and zirconia-reinforced lithium silicate (57.1%). This can be attributed to the high stiffness and brittleness of these ceramic materials, which transfer occlusal stresses more aggressively to the underlying tooth structure. The monolithic nature of the restorations, combined with their high elastic modulus, limits their ability to absorb functional stresses, causing fracture lines to propagate beyond the restoration into the root dentin. Similar findings were reported by Nasr et al. (2025) and Al-Zordk et al. (2021), who observed increased catastrophic failures in brittle, high-strength ceramics bonded to dentin, especially under compressive loading. Additionally, the adhesive interface may act as a stress concentration zone, particularly when internal adaptation is suboptimal or when margins extend into dentin, further promoting root involvement during fracture [64, 65]. 

Conversely, VITA Enamic demonstrated a greater proportion of restorable failures (Types I and II), suggesting more favorable clinical reparability. However, its inconsistent behavior and overall lower fracture threshold diminish its reliability in stress-bearing regions. These results are supported by Guess et al. (2013) and Kruzic et al. (2018), who documented variable failure behavior and fatigue performance in hybrid ceramics [33, 66]. 

Notably, a recent study by Nasr et al. (2025) corroborates the superior fatigue resistance of high-translucent zirconia and lithium disilicate over hybrid ceramics, particularly under complex loading and environmental conditions [67]. Stress accumulation at the adhesive interface remains a concern for PICNs, especially when used in thin sections or under high occlusal forces.

Despite the differences in performance, all tested materials exhibited fracture resistance values exceeding the physiological occlusal load in premolars, which is typically estimated at 400–1000 N depending on functional and parafunctional conditions [68, 69]. This supports the clinical viability of all four materials under controlled circumstances. However, Zolid FX and IPS e.max CAD demonstrated broader safety margins, making them more suitable for high-stress posterior applications.

While the elastic modulus of VITA Enamic may help dissipate forces, it may also contribute to concentrated stresses at the adhesive interface, paradoxically increasing the risk of catastrophic failure. As suggested by Huang et al. (2020), proper preparation design with rounded internal contours plays a crucial role in mitigating stress and reducing the severity of failure in overlay restorations [70]. 

Although VITA Enamic may offer intraoral repair potential due to its flexible structure, the presence of catastrophic fractures involving the tooth structure in this study limits its use in high-load cases. These results are consistent with findings from Zimmermann et al. (2017), Sieper et al. (2017), and Naffah et al. (2019), who observed similar failure trends in hybrid ceramics following fatigue testing [53, 71, 72]. Thus, careful clinical judgment is essential when selecting hybrid ceramics for posterior restorations.

Recent evidence by Mihali and Hiller (2025) has emphasized that the mechanical behavior of CAD/CAM restorative materials cannot be interpreted solely based on fracture resistance values. Their findings highlighted significant differences in the structural and biomechanical performance of zirconia, glass-ceramics, and hybrid materials, reflecting variations in their internal microstructure and composition. This supports the concept that each material class exhibits a distinct balance between stiffness, strength, and energy absorption capacity, which directly influences its clinical performance under functional loading conditions [73]. 

Although zirconia and lithium disilicate demonstrated higher fracture resistance values, they were predominantly associated with catastrophic failure modes, which are clinically unfavorable due to their non-repairable nature. In contrast, materials with lower fracture resistance showed more favorable, repairable failure patterns, which may allow for conservative clinical management. Therefore, evaluation of restorative materials should consider not only the magnitude of fracture resistance but also the nature of failure and its clinical implications [74]. 

The findings of the present study should not be interpreted solely based on fracture resistance values. Although zirconia and lithium disilicate exhibited higher fracture resistance, their failure patterns were predominantly catastrophic and non-repairable, which may limit their clinical advantage in certain situations. In contrast, materials with lower fracture resistance tended to demonstrate more favorable and potentially repairable failure modes. Accordingly, these results highlight an important clinical trade-off between mechanical strength and failure behavior, suggesting that restorative material selection should be guided not only by resistance to fracture but also by the mode and clinical consequences of failure. As supported by Vervack et al. 2024 and Magne & Belser, 2003 [74, 75]. 

It is important to acknowledge the limitations of this study. Despite simulating masticatory function via cyclic loading, other intraoral variables such as saliva, temperature fluctuations, and acidic pH were not replicated. Furthermore, loading was applied in a single axial direction, which does not fully represent the multidirectional forces present in the oral cavity. Future studies should include thermocycling and long-term clinical follow-up to validate in vitro outcomes.

Long-term clinical evidence reported by Loloș et al. (2025) demonstrated high survival rates of zirconia-based restorations, particularly monolithic zirconia, which exhibited excellent durability and overall clinical success over extended follow-up periods. Despite variations in failure modes observed in laboratory studies, their clinical data indicated that zirconia restorations maintain high performance in functionally demanding environments, supporting their reliability as a restorative material in posterior load-bearing regions [76]. 

Preliminary clinical investigations, such as the study by Hazzaa et al. (2023), found no significant differences between vonlay and onlay restorations over a one-year period based on USPHS criteria [77]. These findings support the clinical applicability of vonlay restorations when properly designed and bonded.

It is important to interpret the findings of the present study within the inherent limitations of in vitro investigations. While the results provide valuable comparative insights under standardized laboratory conditions, they do not directly reflect long-term clinical performance. Therefore, caution should be exercised when extrapolating these outcomes to clinical scenarios, as multiple intraoral factors—such as fatigue loading, thermal fluctuations, and biological variability—may significantly influence material behavior over time.

Accordingly, the present findings should be considered as indicative of relative material performance rather than definitive predictors of clinical success.

## Conclusion

In conclusion, this study confirms that the choice of CAD/CAM material significantly affects the biomechanical performance of vonlay restorations. Zirconia and lithium disilicate materials offered superior fracture resistance and more predictable failure behavior, whereas polymer-infiltrated ceramics displayed greater variability and lower reliability in stress-bearing regions.

Within the limitations of this in vitro study, the findings demonstrate that Material selection should consider the balance between fracture resistance and failure mode, rather than relying solely on mechanical strength.

Super-high-translucent zirconia and lithium disilicate exhibited the highest fracture resistance and more predictable—but catastrophic—failure modes.

Zirconia-reinforced lithium silicate displayed intermediate mechanical performance, with fracture resistance and failure patterns comparable to lithium disilicate in most cases.

Polymer-infiltrated ceramic demonstrated the lowest fracture resistance and a greater incidence of restorable failure modes.

All tested materials exceeded physiological occlusal load thresholds, but their distinct mechanical behaviors emphasize the importance of case-specific material selection based on functional and esthetic demands, these findings should be interpreted with caution, as in vitro results do not necessarily reflect long-term clinical performance.

Further long-term clinical investigations and fatigue studies under thermomechanical cycling are necessary to validate these findings and to determine the most appropriate indications for each material in daily clinical practice. Future studies are recommended to incorporate a reference control groups alongside different preparation designs and long-term fatigue loading. Such approaches may provide a more comprehensive understanding of the mechanical performance and clinical behavior of CAD/CAM materials.

## Supplementary Information

Supplementary Material 1.

## Data Availability

The datasets generated and/or analyzed during the current study are available from the corresponding author on reasonable request.

## Abbreviations

10-MDP: 10-Methacryloyloxydecyl Dihydrogen Phosphate
BPDM: Biphenyl dimethacrylate
CAD-CAM: Computer aided design and computer aided manufacturing
EM: E-max Cad
LDCs: Lithium Disilicate
min: Minute
MMA: Methyl methacrylate
N: Newton
PMMA: Polymethylmethacrylate

## References

[1] Blatz MB. Long-term clinical success of all-ceramic posterior restorations. Quintessence Int. 2002;33:415–26. 12073722

[2] Blatz MB, Chiche G, Bahat O, Roblee R, Coachman C, Heymann HO. Evolution of aesthetic dentistry. J Dent Res. 2019;98:1294–304. 10.1177/0022034519875450. 31633462 10.1177/0022034519875450

[3] Jurado CA, Villalobos-Tinoco J, Tsujimoto A, Castro P, Torrealba Y. The art of minimal tooth reduction for veneer restorations. Eur J Gen Dent. 2020;9:45–52. 10.4103/ejgd.ejgd_173_19.

[4] Douglass CW, Sheets CG. Patients’ expectations for oral health care in the 21st century. J Am Dent Assoc. 2000;131:S3–7. 10.14219/jada.archive.2000.0397. 10.14219/jada.archive.2000.0397 10860338

[5] Felden A, Schmalz G, Federlin M, Hiller KA. Retrospective clinical investigation and survival analysis on ceramic inlays and partial ceramic crowns: results up to 7 years. Clin Oral Investig. 1998;2:161–7. 10.1007/s007840050064. 10388388 10.1007/s007840050064

[6] McLaren EA, Figueira J, Goldstein RE. Vonlays: a conservative esthetic alternative to full-coverage crowns. Compend Contin Educ Dent. 2015;36:282–9. 25821940

[7] Nadig RR, Garian R. Stress distribution pattern in endodontically treated mesio occlusally involved premolars restored with ceramic onlay, vonlay and full crown restorations. IOSR J Dent Med Sci. 2022;21:58–66. 10.9790/0853-2105055866.

[8] Mookhtiar H, Vonlay. A Paradigm Shift in Post Endodontic Restoration: A Case Report. Ann Clin Med Case Rep. 2022;9:1–4.

[9] Veneziani M. Posterior indirect adhesive restorations: updated indications and the Morphology Driven Preparation Technique. Int J Esthet Dent. 2017;12:204–30. 28653051

[10] Edelhoff D, Güth JF, Erdelt K, Brix O, Liebermann A. Clinical performance of occlusal onlays made of lithium disilicate ceramic in patients with severe tooth wear up to 11 years. Dent Mater. 2019;35:1319–30. 10.1016/j.dental.2019.06.001. 31256912 10.1016/j.dental.2019.06.001

[11] Sayed E, Waz S, Mohamed M. Influence of Different Vonlay Preparation Designs on Fracture Resistance. Egypt Dent J. 2022;68:2733–41. 10.21608/edj.2022.140789.2122.

[12] Suresh C, Kommi V. CAD/CAM Ceramics-The Paradigm Shift in Fixed Prosthodontics. Eur J Mol Clin Med. 2020;7:2118–26.

[13] Li RW, Chow TW, Matinlinna JP. Ceramic dental biomaterials and CAD/CAM technology: state of the art. J Prosthodont Res. 2014;58:208–16. 10.1016/j.jpor.2014.07.003. 25172234 10.1016/j.jpor.2014.07.003

[14] Alberto Jurado C, Kaleinikova Z, Tsujimoto A, Alberto Cortés Treviño D, Seghi RR, Lee DJ. Comparison of Fracture Resistance for Chairside CAD/CAM Lithium Disilicate Crowns and Overlays with Different Designs. J Prosthodont. 2022;31:341–7. 10.1111/jopr.13411. 34297866 10.1111/jopr.13411

[15] Luciano M, Francesca Z, Michela S, Tommaso M, Massimo A. Lithium disilicate posterior overlays: clinical and biomechanical features. Clin Oral Investig. 2020;24:841–8. 10.1007/s00784-019-02972-3. 31201516 10.1007/s00784-019-02972-3 PMC13161036

[16] Clausen JO, Abou Tara M, Kern M. Dynamic fatigue and fracture resistance of non-retentive all-ceramic full-coverage molar restorations. Influence of ceramic material and preparation design. Dent Mater. 2010;26:533–8. 10.1016/j.dental.2010.01.011. 20181388 10.1016/j.dental.2010.01.011

[17] Krummel A, Garling A, Sasse M, Kern M. Influence of bonding surface and bonding methods on the fracture resistance and survival rate of full-coverage occlusal veneers made from lithium disilicate ceramic after cyclic loading. Dent Mater. 2019;35:1351–9. 10.1016/j.dental.2019.07.001. 31351579 10.1016/j.dental.2019.07.001

[18] Ma L, Guess PC, Zhang Y. Load-bearing properties of minimal-invasive monolithic lithium disilicate and zirconia occlusal onlays: finite element and theoretical analyses. Dent Mater. 2013;29:742–51. 10.1016/j.dental.2013.04.004. 23683531 10.1016/j.dental.2013.04.004 PMC3698988

[19] Elsayed M, Sherif R, El-khodary N. Fracture resistance of Vita suprinity versus IPS e. max CAD vonlays restoring premolars (An in vitro study). Int J Appl Dent Sci. 2020;6:734–41. 10.22271/oral.2020.v6.i3k.1029.

[20] Traini T, Sinjari B, Pascetta R, Serafini N, Perfetti G, Trisi P, et al. The Zirconia – Reinforced Lithium Silicate Ceramic: Lights and Shadows of a New Material. Dent Mater J. 2016;35:748–55. 10.4012/dmj.2016-041. 27546858 10.4012/dmj.2016-041

[21] Elsaka SE, Elnaghy AM. Mechanical properties of zirconia reinforced lithium silicate glass-ceramic. Dent Mater. 2016;32:908–14. 10.1016/j.dental.2016.03.013. 27087687 10.1016/j.dental.2016.03.013

[22] Larsson C, Wennerberg A. The clinical success of zirconia-based crowns: a systematic review. Int J Prosthodont. 2014;27:33–43. 10.11607/ijp.3647. 24392475 10.11607/ijp.3647

[23] Özkurt Z, Kazazoğlu E. Clinical success of zirconia in dental applications. J Prosthodont. 2010;19:64–8. 10.1111/j.1532-849X.2009.00513.x. 19754642 10.1111/j.1532-849X.2009.00513.x

[24] Guess PC, Bonfante EA, Silva NR, Coelho PG, Thompson VP. Effect of core design and veneering technique on damage and reliability of Y-TZP-supported crowns. Dent Mater. 2013;29:307–16. 10.1016/j.dental.2012.11.012. 23228337 10.1016/j.dental.2012.11.012

[25] Zhang Y. Making yttria-stabilized tetragonal zirconia translucent. Dent Mater. 2014;30:1195–203. 10.1016/j.dental.2014.08.375. 25193781 10.1016/j.dental.2014.08.375 PMC4167579

[26] Alammar A, Blatz MB. The Resin Bond to High Translucent Zirconia- A Systematic Review. J Esthetic Rest Dent. 2022;34:117–35. 10.1111/jerd.12876. 10.1111/jerd.12876 35072329

[27] Harsha MS, Praffulla M, Babu MR, Leneena G, Krishna TS, Divya G. The effect of cavity design on fracture resistance and failure pattern in monolithic zirconia partial coverage restorations-an in vitro study. J Clin Diagn Res. 2017;11:ZC45–8. 10.7860/JCDR/2017/25305.9856. 28658906 10.7860/JCDR/2017/25305.9856 PMC5483808

[28] Zhang F, Reveron H, Spies BC, Van Meerbeek B, Chevalier J. Trade-off between fracture resistance and translucency of zirconia and lithium disilicate glass ceramics for monolithic restorations. Acta Biomater. 2019;91:24–34. 10.1016/j.actbio.2019.04.043. 31034947 10.1016/j.actbio.2019.04.043

[29] Gürpinar B, Çelakil T, Baca E, Evlioğlu G. Fracture resistance of occlusal veneer and overlay CAD/CAM restorations made of polymer-infiltrated ceramic and lithium disilicate ceramic blocks. Ege Üniversitesi Diş Hekimliği Fakültesi. 2020;41:131–42. 10.5505/eudfd.2020.85866.

[30] Al-Akhali M, Chaar MS, Elsayed A, Samran A, Kern M. Fracture resistance of ceramic and polymer-based occlusal veneer restorations. J Mech Behav Biomed Mater. 2017;74:245–50. 10.1016/j.jmbbm.2017.06.013. 28633093 10.1016/j.jmbbm.2017.06.013

[31] Schlichting LH, Maia HP, Baratieri LN, Magne P. Novel-design ultra-thin CAD/CAM composite resin and ceramic occlusal veneers for the treatment of severe dental erosion. J Prosthet Dent. 2011;105:217–26. 10.1016/S0022-3913(11)60035-8. 21458646 10.1016/S0022-3913(11)60035-8

[32] Boukhris H, Touffeha G, M’ghirbi N, Karoui L, Hajjami H. Veneerlays: A suitable Conservative Approach for Restoring Posterior Teeth. J Dent Med Sci Res. 2018;2:15–9.

[33] Guess PC, Schultheis S, Wolkewitz M, Zhang Y, Strub JR. Influence of preparation design and ceramic thicknesses on fracture resistance and failure modes of premolar partial coverage restorations. J Prosthet Dent. 2013;110:264–73. 10.1016/S0022-3913(13)60374-1. 24079561 10.1016/S0022-3913(13)60374-1 PMC4449616

[34] Nelson SJ. WHEELER’S Dental Anatomy, Physiology, and Occlusion. 10th ed. St. Louis, Missouri: Elsevier; 1974.

[35] Emir F, Ayyildiz S, Sahin C. What is the changing frequency of diamond burs? J Adv Prosthodont. 2018;10:93–100. 10.4047/jap.2018.10.2.93. 29713429 10.4047/jap.2018.10.2.93 PMC5917112

[36] Aboushelib MN. Fatigue and fracture resistance of zirconia crowns prepared with different finish line designs. J Prosthodont. 2012;21:22–7. 10.1111/j.1532-849X.2011.00787.x. 22040309 10.1111/j.1532-849X.2011.00787.x

[37] Papia E, Larsson C, du Toit M, von Steyern PV. Bonding between oxide ceramics and adhesive cement systems: a systematic review. J Biomed Mater Res B Appl Biomater. 2014;102:395–413. 10.1002/jbm.b.33013. 24123837 10.1002/jbm.b.33013

[38] Scherrer SS, Cesar PF, Swain MV. Direct comparison of the bond strength results of the different test methods: a critical literature review. Dent Mater. 2010;26:e78–93. 10.1016/j.dental.2009.12.002. 20060160 10.1016/j.dental.2009.12.002

[39] Thompson JY, Stoner BR, Piascik JR, Smith R. Adhesion/cementation to zirconia and other non-silicate ceramics: where are we now? Dent Mater. 2011;27:71–82. 10.1016/j.dental.2010.10.022. 21094526 10.1016/j.dental.2010.10.022 PMC3046396

[40] Wang RR, Lu CL, Wang G, Zhang DS. Influence of cyclic loading on the fracture toughness and load bearing capacities of all-ceramic crowns. Int J Oral Sci. 2014;6:99–104. 10.1038/ijos.2013.94. 24335786 10.1038/ijos.2013.94 PMC5130053

[41] Baladhandayutham B, Lawson NC, Burgess JO. Fracture load of ceramic restorations after fatigue loading. J Prosthet Dent. 2015;114:266–71. 10.1016/j.prosdent.2015.03.006. 25985741 10.1016/j.prosdent.2015.03.006

[42] Lawson NC, Jurado CA, Huang CT, Morris GP, Burgess JO, Liu PR, et al. Effect of surface treatment and cement on fracture load of traditional zirconia (3Y), translucent zirconia (5Y), and lithium disilicate crowns. J Prosthodont. 2019;28:659–65. 10.1111/jopr.13088. 31145492 10.1111/jopr.13088 PMC6642729

[43] Elsaka SE, Elnaghy AM. Effect of Surface Treatment and Aging on Bond Strength of Composite Cement to Novel CAD/CAM Nanohybrid Composite. J Adhes Dent. 2020;22:195–204. 10.3290/j.jad.a44284. 32322840 10.3290/j.jad.a44284

[44] Lin WS, Ercoli C, Feng C, Morton D. The effect of core material, veneering porcelain, and fabrication technique on the biaxial flexural strength and weibull analysis of selected dental ceramics. J Prosthodont. 2012;21:353–62. 10.1111/j.1532-849X.2012.00845.x. 22462639 10.1111/j.1532-849X.2012.00845.x

[45] Kim JH, Cho J, Lee Y, Cho BH. The Survival of Class V Composite Restorations and Analysis of Marginal Discoloration. Oper Dent. 2017;42:E93–101. 10.2341/16-186-C. 28467254 10.2341/16-186-C

[46] Al-Akhali M, Kern M, Elsayed A, Samran A, Chaar MS. Influence of thermomechanical fatigue on the fracture strength of CAD-CAM-fabricated occlusal veneers. J Prosthet Dent. 2019;121:644–50. 10.1016/j.prosdent.2018.07.019. 30711291 10.1016/j.prosdent.2018.07.019

[47] Willard A, Gabriel Chu TM. The science and application of IPS e.Max dental ceramic. Kaohsiung J Med Sci. 2018;34:238–42. 10.1016/j.kjms.2018.01.012. 29655413 10.1016/j.kjms.2018.01.012 PMC11915628

[48] Morimoto S, Rebello de Sampaio FB, Braga MM, Sesma N, Özcan M. Survival rate of resin and ceramic inlays, onlays, and overlays: A systematic review and meta-analysis. J Dent Res. 2016;95:985–94. 10.1177/0022034516652848. 27287305 10.1177/0022034516652848

[49] Czechowski Ł, Dejak B, Konieczny B, Krasowski M. Evaluation of fracture resistance of occlusal veneers made of different types of materials depending on their thickness. Mater (Basel). 2023;16:6006. 10.3390/ma16176006. 10.3390/ma16176006 PMC10489006 37687699

[50] Sadeqi HA, Baig MR, Al-Shammari M. Evaluation of marginal/internal fit and fracture load of monolithic zirconia and zirconia lithium silicate (ZLS) CAD/CAM crown systems. Mater (Basel). 2021;14:6346. 10.3390/ma14216346. 10.3390/ma14216346 PMC8585271 34771872

[51] Sulaiman TA, Delgado AJ, Donovan TE. Survival rate of lithium disilicate restorations at 4 years: A retrospective study. J Prosthet Dent. 2015;114:364–6. 10.1016/j.prosdent.2015.04.011. 26050028 10.1016/j.prosdent.2015.04.011

[52] Al-Zordk W, Saudi A, Abdelkader A, Taher M, Ghazy M. Fracture resistance and failure mode of mandibular molar restored by occlusal veneer: Effect of material type and dental bonding surface. Mater (Basel). 2021;14:6476. 10.3390/ma14216476. 10.3390/ma14216476 PMC8585144 34772003

[53] Zimmermann M, Egli G, Zaruba M, Mehl A. Influence of material thickness on fractural strength of CAD/CAM fabricated ceramic crowns. Dent Mater J. 2017;36:778–83. 10.4012/dmj.2016-296. 28835598 10.4012/dmj.2016-296

[54] Coldea A, Swain MV, Thiel N. In-vitro strength degradation of dental ceramics and novel PICN material by sharp indentation. J Mech Behav Biomed Mater. 2013;26:34–42. 10.1016/j.jmbbm.2013.05.004. 23807311 10.1016/j.jmbbm.2013.05.004

[55] Apel E, van’t Hoen C, Rheinberger V, Höland W. Influence of ZrO2 on the crystallization and properties of lithium disilicate glass-ceramics derived from a multi-component system. J Eur Ceram Soc. 2007;27:1571–7. 10.1016/j.jeurceramsoc.2006.04.103.

[56] Hikita K, Van Meerbeek B, De Munck J, Ikeda T, Van Landuyt K, Maida T, et al. Bonding effectiveness of adhesive luting agents to enamel and dentin. Dent Mater. 2007;23:71–80. 10.1016/j.dental.2005.12.002. 16426673 10.1016/j.dental.2005.12.002

[57] Yildiz C, Vanlıoğlu BA, Evren B, Uludamar A, Kulak- Ozkan Y. Fracture resistance of manually and CAD/CAM manufactured ceramic onlays. J Prosthodont. 2013;22:537–42. 10.1111/jopr.12037. 23758595 10.1111/jopr.12037

[58] Preis V, Behr M, Hahnel S, Rosentritt M. Influence of cementation on in vitro performance, marginal adaptation and fracture resistance of CAD/CAM fabricated ZLS molar crowns. Dent Mater. 2015;31:1363–9. 10.1016/j.dental.2015.08.154. 26345998 10.1016/j.dental.2015.08.154

[59] Hamza TA, Sherif RM. Fracture resistance of monolithic Glass-ceramics Versus Bilayered Zirconia-based restorations. J Prosthodont. 2019;28:e259–64. 10.1111/jopr.12684. 29044828 10.1111/jopr.12684

[60] Abdelaal AM, Kehela HA, Holiel AA. Fracture resistance and fractographic analysis of pressable glass-ceramics with different partial coverage designs for maxillary premolars. BMC Oral Health. 2024;24:1078. 10.1186/s12903-024-04809-2. 39272065 10.1186/s12903-024-04809-2 PMC11395659

[61] Fayed AK, Azer AS, AboElhassan RG. Fit accuracy and fracture resistance evaluation of advanced lithium disilicate crowns (in- vitro study). BMC Oral Health. 2025;25:58. 10.1186/s12903-024-05325-z. 39799312 10.1186/s12903-024-05325-z PMC11725217

[62] Andrade JP, Stona D, Bittencourt HR, Borges GA, Burnett LH, Júnior, Spohr AM. Effect of different computer-aided design/computer-aided manufacturing (CAD/CAM) materials and thicknesses on the fracture resistance of occlusal veneers. Oper Dent. 2018;43:539–48. 10.2341/17-131-L. 29513638 10.2341/17-131-L

[63] Petrini M, Ferrante M, Su B. Fabrication and characterization of biomimetic ceramic/polymer composite materials for dental restoration. Dent Mater. 2013;29:375–81. 10.1016/j.dental.2012.12.004. 23305963 10.1016/j.dental.2012.12.004

[64] Al-Zordk W, Saudi A, Abdelkader A, Taher M, Ghazy M. Fracture resistance and failure mode of mandibular molar restored by occlusal veneer: Effect of material type and dental bonding surface. Materials. 2021;14:6476. 34772003 10.3390/ma14216476 PMC8585144

[65] Nasr DM, AboElHassan RG. Effect of different ceramic materials and dentin sealing on occlusal veneers bond strength and fracture resistance. BMC Oral Health. 2025;25:186. 10.1186/s12903-025-05505-5. 39905379 10.1186/s12903-025-05505-5 PMC11796217

[66] Kruzic JJ, Arsecularatne JA, Tanaka CB, Hoffman MJ, Cesar PF. Recent advances in understanding the fatigue and wear behavior of dental composites and ceramics. J Mech Behav Biomed Mater. 2018;88:504–33. 10.1016/j.jmbbm.2018.08.008. 30223214 10.1016/j.jmbbm.2018.08.008

[67] Nasr DM, Abdelraheem IM, Watts DC, Silikas N, Borba M, Alharbi N, et al. Effect of preparation designs and CAD-CAM materials on step-stress fatigue survival of premolar partial coverage restorations: An in-vitro study with fractographic analysis. Dent Mater. 2025;41:122–33. 10.1016/j.dental.2024.11.003. 39616090 10.1016/j.dental.2024.11.003

[68] Bates JF, Staford GD, Harrison A. Masticatory function - a review of the literature. III. Masticatory performance and efficiency. J Oral Rehabil. 1976;3:57–67. 10.1111/j.1365-2842.1976.tb00929.x. 772184 10.1111/j.1365-2842.1976.tb00929.x

[69] Waltimo A, Nyström M, Könönen M. Bite force and dentofacial morphology in men with severe dental attrition. Scand J Dent Res. 1994;102:92–6. 10.1111/j.1600-0722.1994.tb01161.x. 8016561 10.1111/j.1600-0722.1994.tb01161.x

[70] Huang X, Zou L, Yao R, Wu S, Li Y. Effect of preparation design on the fracture behavior of ceramic occlusal veneers in maxillary premolars. J Dent. 2020;97:103346. 10.1016/j.jdent.2020.103346. 32325176 10.1016/j.jdent.2020.103346

[71] Sieper K, Wille S, Kern M. Fracture strength of lithium disilicate crowns compared to polymer-infiltrated ceramic-network and zirconia reinforced lithium silicate crowns. J Mech Behav Biomed Mater. 2017;74:342–8. 10.1016/j.jmbbm.2017.06.025. 28662443 10.1016/j.jmbbm.2017.06.025

[72] Naffah N, Ounsi H, Ozcan M, Bassal H, Salameh Z. Evaluation of the Adaptation and Fracture Resistance of Three CAD-CAM Resin Ceramics: An In vitro Study. J Contemp Dent Pract. 2019;20:571–6. 31316020

[73] Mihali SG, Hiller A. Comparative evaluation of mechanical properties in contemporary prosthetic dental materials: Zirconia, Lithium disilicate, and hybrid composites. Med Evol. 2025;31:320–31.

[74] Vervack V, Johansson C, Coster P, Fokkinga W, Papia E, Vandeweghe S. The fracture strength and the failure mode of lithium disilicate or resin nano ceramics as a crown, overlay, or endocrown restoration on endodontically treated teeth. J Esthet Restor Dent. 2024;36:796–803. 10.1111/jerd.13187. 38152852 10.1111/jerd.13187

[75] Magne P, Belser UC. Porcelain versus composite inlays/onlays: effects of mechanical loads on stress distribution, adhesion, and crown flexure. Int J Periodontics Restor Dent. 2003;23:543–55. 14703758

[76] Lolos D, Mihali SG, Dinu S, Mitariu M, Tudor A, Oancea R. Retrospective long-term survival rate and clinical performance of zirconium oxide restorations over the past 5 years: A comparative study between single crowns and fixed dental prostheses. Med (Kaunas). 2025;61:210. 10.3390/medicina61020210. 10.3390/medicina61020210 PMC11857140 40005327

[77] Hazzaa MA, El Mahallawi OS, Anwar E, Badran A. Clinical outcomes of premolars restored with ceramic vonlay restorations versus onlay using modified USPHS criteria (Randomized Clinical Trial). J Pharm Negat Results. 2023;1010–21. 10.47750/pnr.2023.14.03.132.

