# Supplementary material for: Fracture resistance of ceramic vonlays fabricated from different CAD/CAM materials restoring premolars after cyclic loading (an in vitro study)
Source: BMC Oral Health. 2026 May 9;26:879. doi: 10.1186/s12903-026-08493-2 (PMC13191926; doi:10.1186/s12903-026-08493-2)
Supplement: Supplementary file 1 — Supplementary Material 1. [file 12903_2026_8493_MOESM1_ESM.docx]

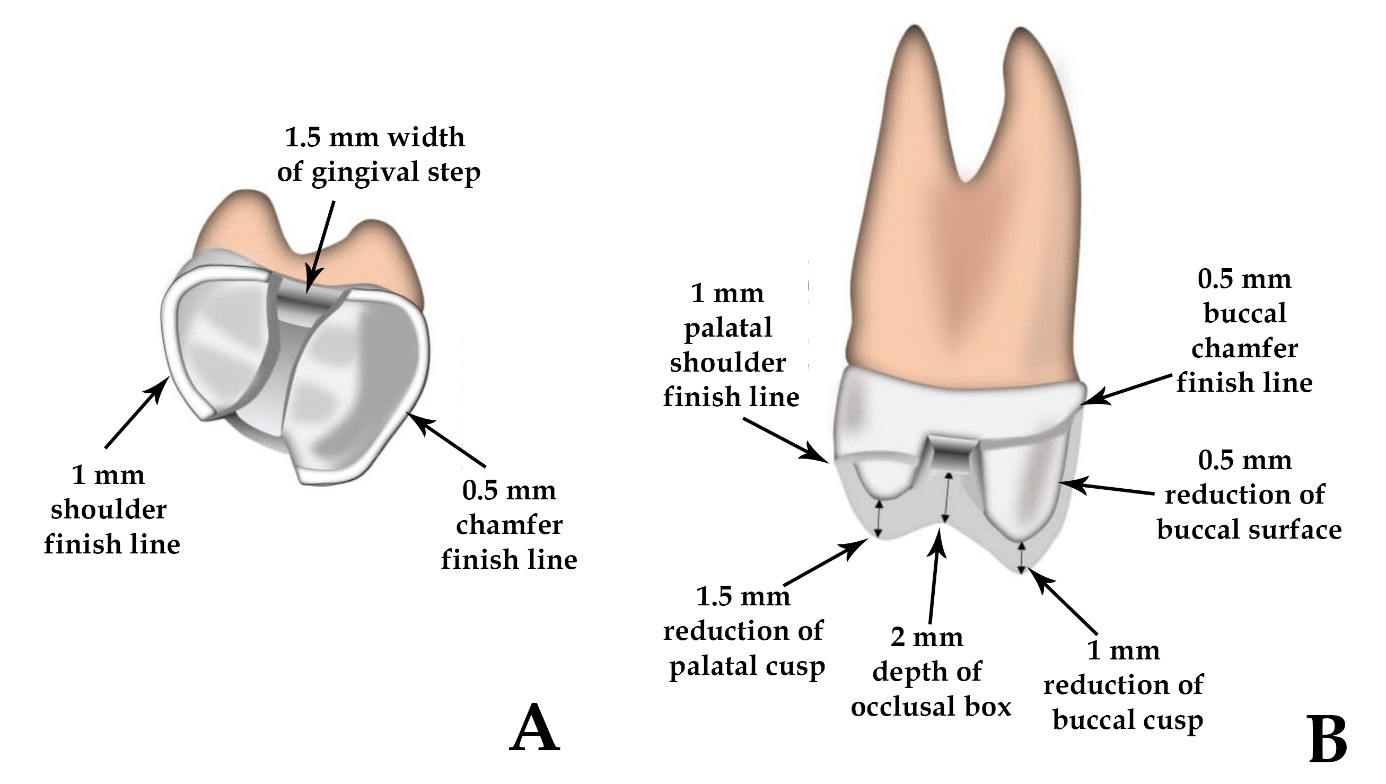


**Figure 1(A-B):** Illustrations showing, Unprocessed and uncropped original blot images are provided as Supplementary Data. Each blot corresponds to the figures presented in the main manuscript and includes all lanes, including those not shown in the final figure. Molecular weight markers are visible, and no selective adjustments were made. The data are presented in their original form to ensure transparency, reproducibility, and integrity of the experimental results.

| 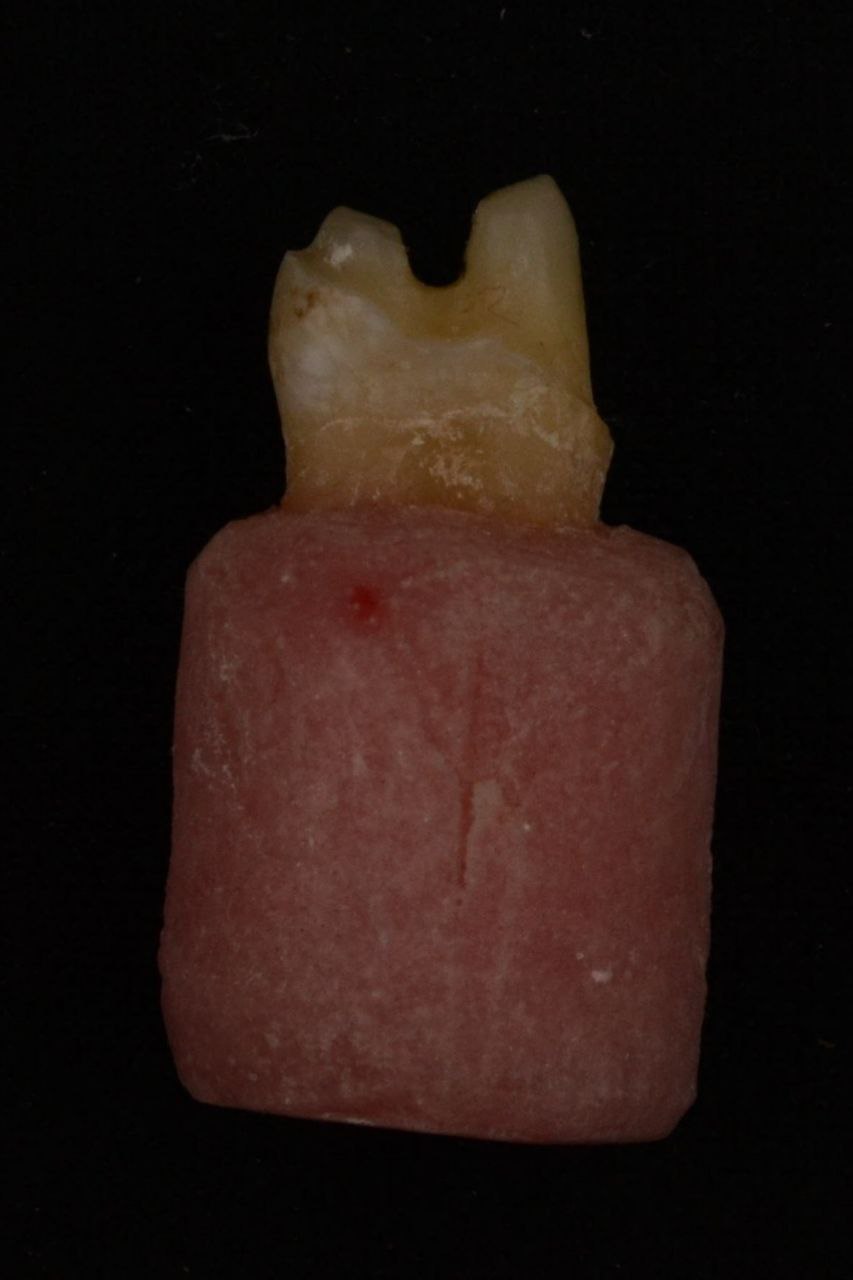 | 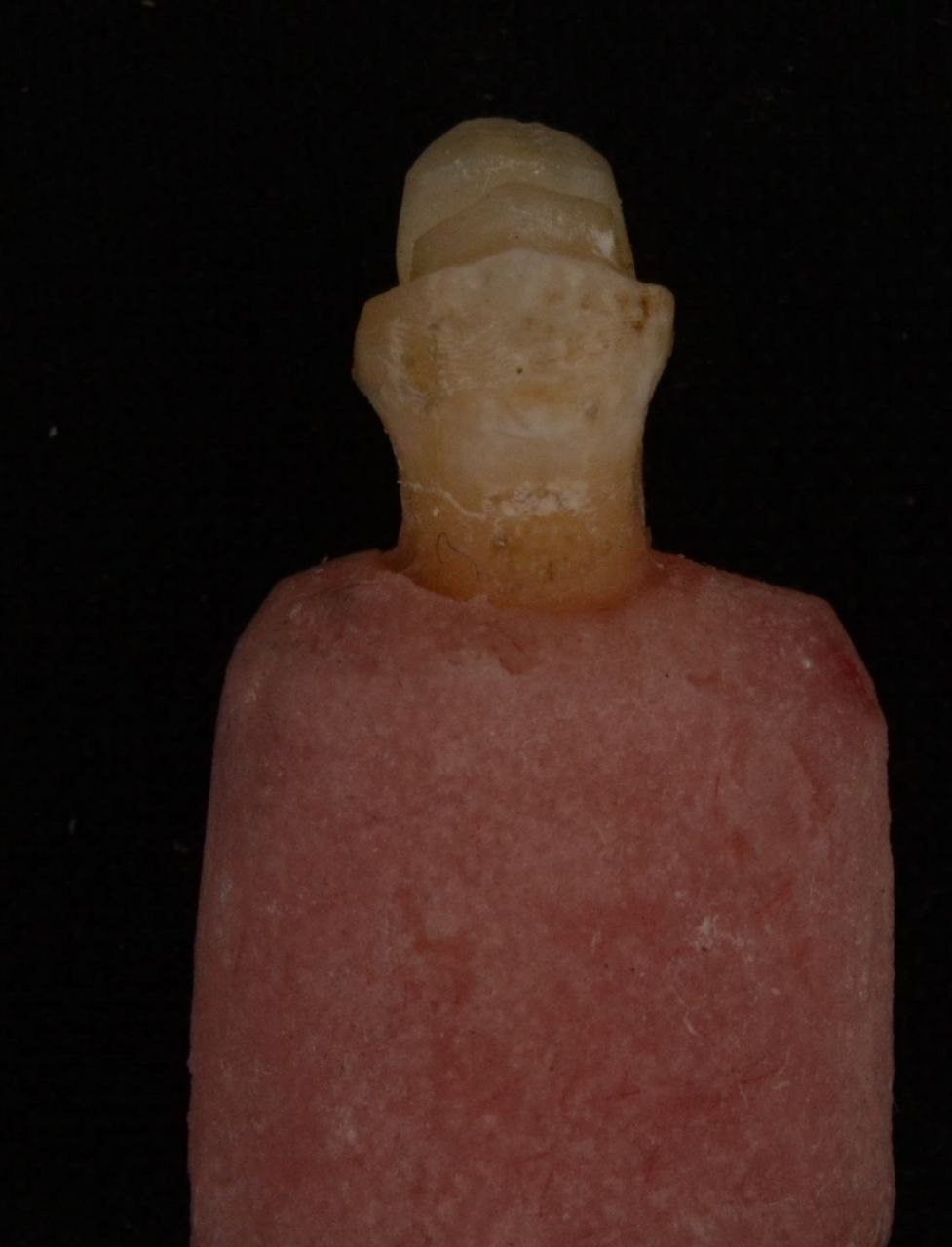 |
| --- | --- |

**Figure 2(a-b):** Full, uncropped image corresponding to the experimental setup. This figure shows the complete structure including all relevant anatomical or experimental details. No modifications were applied to the image beyond uniform contrast enhancement. All visible regions are retained to ensure reproducibility and transparency.


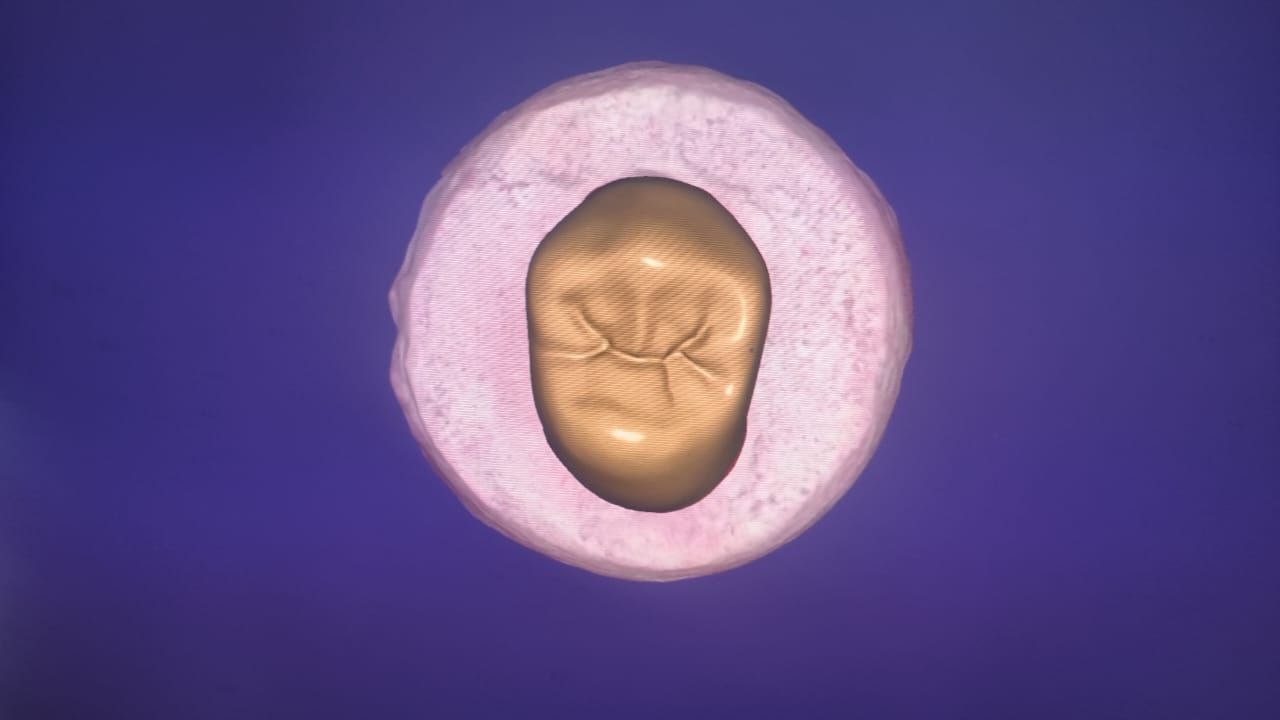


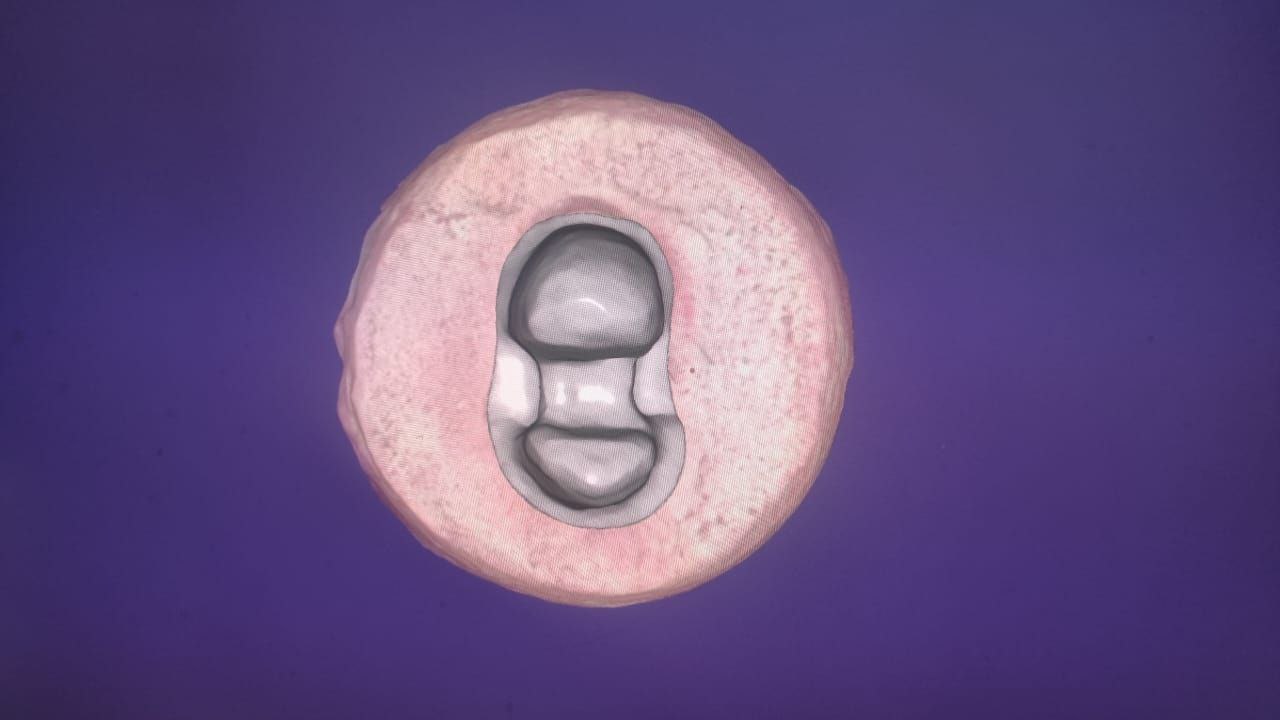


**Figure 3****(a-b):** Full, uncropped image corresponding to the experimental setup.

This figure shows the complete structure including all relevant anatomical or experimental details. No modifications were applied to the image beyond uniform contrast enhancement. All visible regions are retained to ensure reproducibility and transparency.


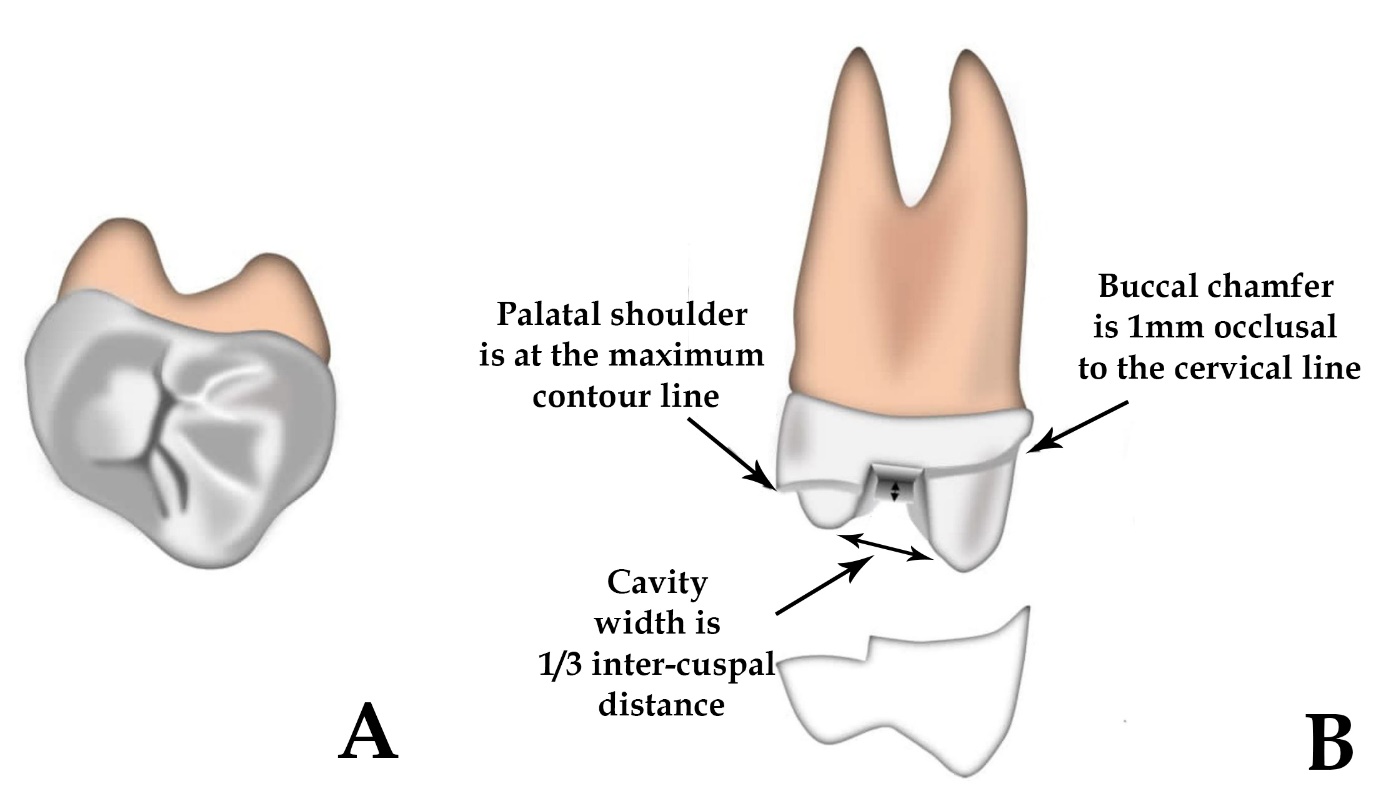


**Figure 4(A-B):** Illustrations showing, Unprocessed and uncropped original blot images are provided as Supplementary Data. Each blot corresponds to the figures presented in the main manuscript and includes all lanes, including those not shown in the final figure. Molecular weight markers are visible, and no selective adjustments were made. The data are presented in their original form to ensure transparency, reproducibility, and integrity of the experimental results.


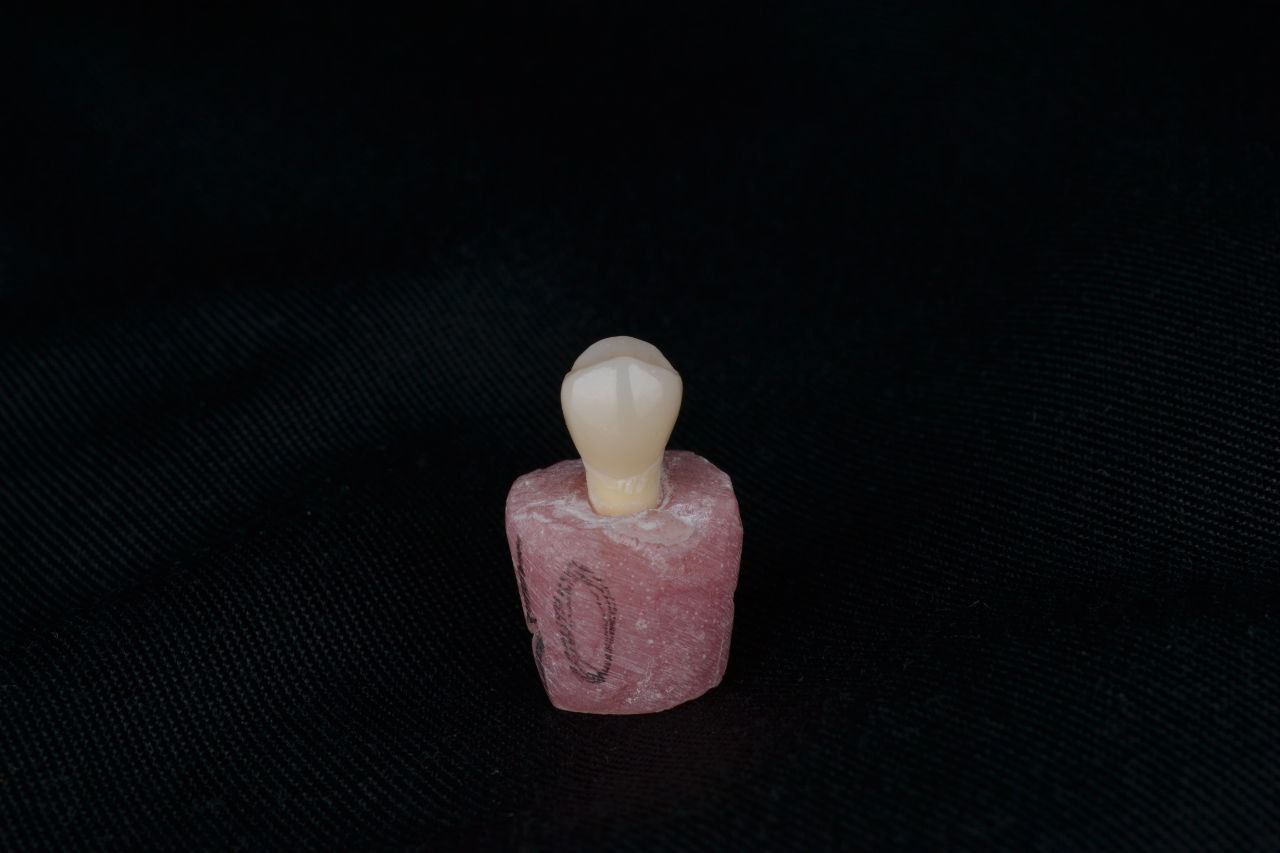


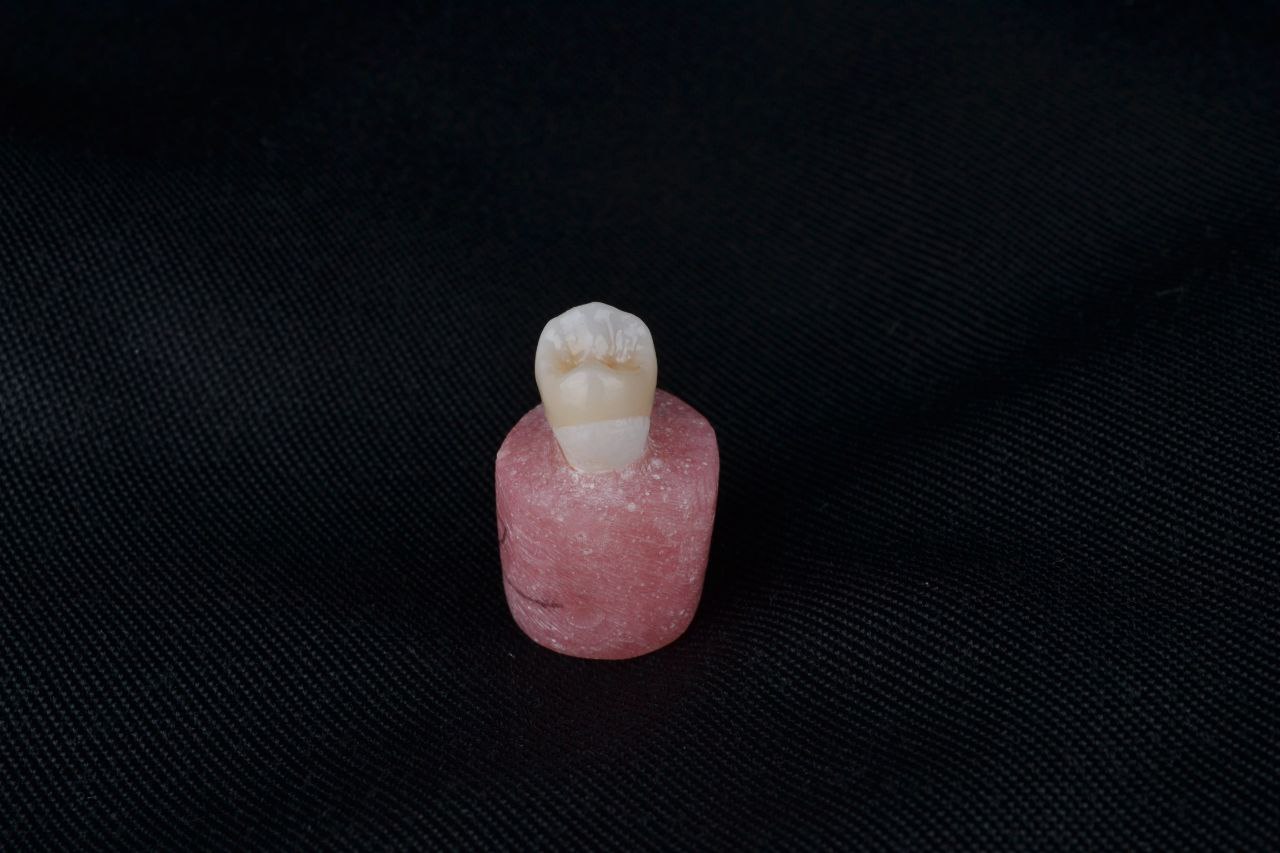


**Figure 5(a-b):** Cemented vonlay (a) buccal view (b) palatal view.

Full, uncropped image corresponding to the experimental setup. This figure shows the complete structure including all relevant anatomical or experimental details. No modifications were applied to the image beyond uniform contrast enhancement. All visible regions are retained to ensure reproducibility and transparency.


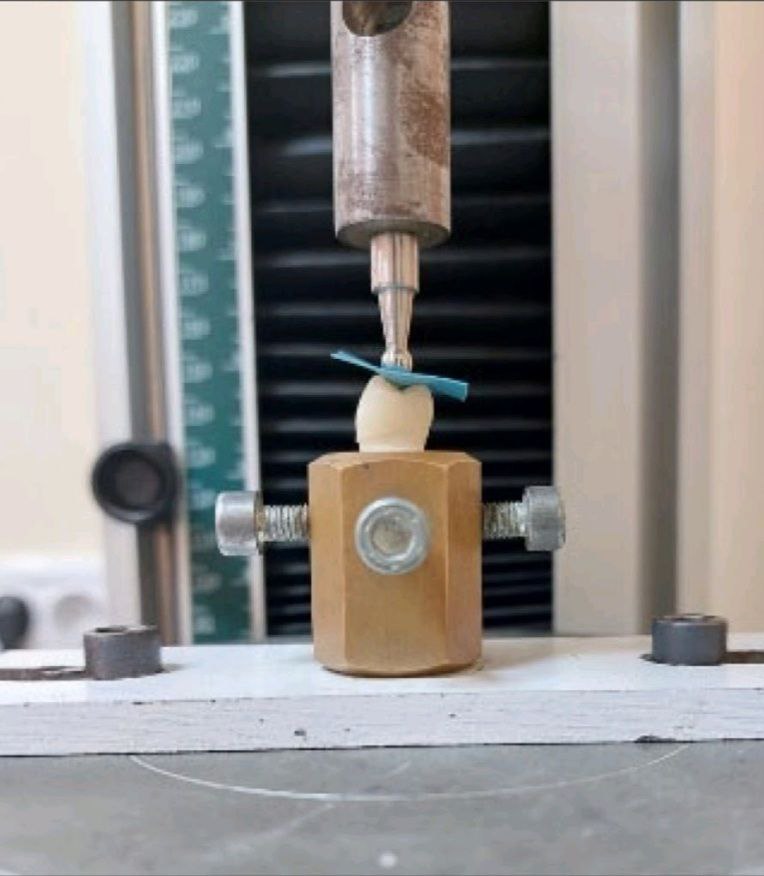


**Figure 6:** Fracture resistance test. (Tinius Olsen Model 5ST, Tinius Olsen Ltd., England, 2018)

Full, uncropped image corresponding to the experimental setup. This figure shows the complete structure including all relevant anatomical or experimental details. No modifications were applied to the image beyond uniform contrast enhancement. All visible regions are retained to ensure reproducibility and transparency.


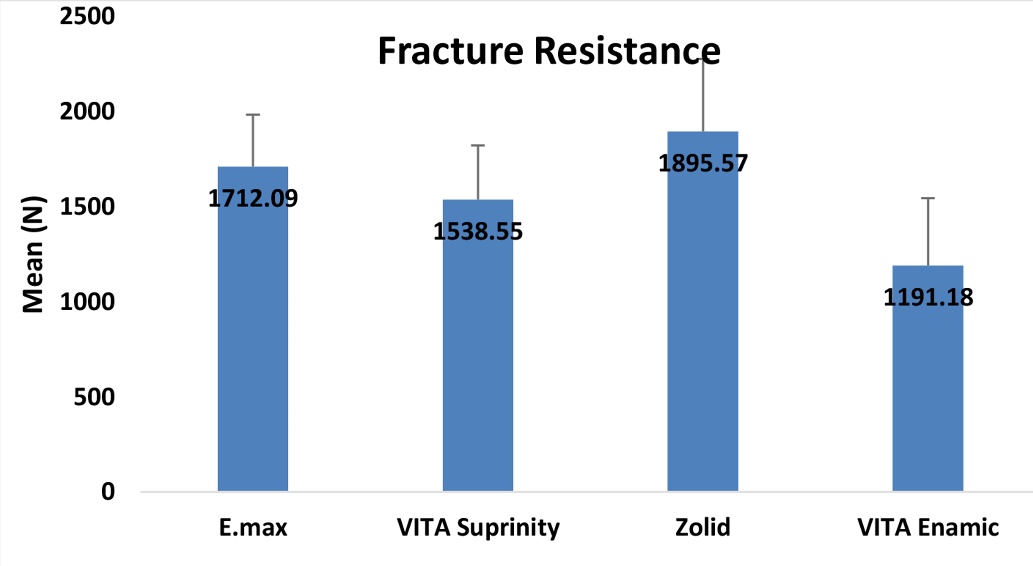


**Figure 7:** Chart showing a comparison of fracture resistance between four studied groups.

Illustrations showing, Unprocessed and uncropped original blot images are provided as Supplementary Data. Each blot corresponds to the figures presented in the main manuscript and includes all lanes, including those not shown in the final figure. Molecular weight markers are visible, and no selective adjustments were made. The data are presented in their original form to ensure transparency, reproducibility, and integrity of the experimental results.

After *fracture resistance test*, all the fractured surfaces of Vonlay restorations were carefully examined at **20x magnification** to identify the *failure mode* using **Stereomicroscope** and they were classified into four types


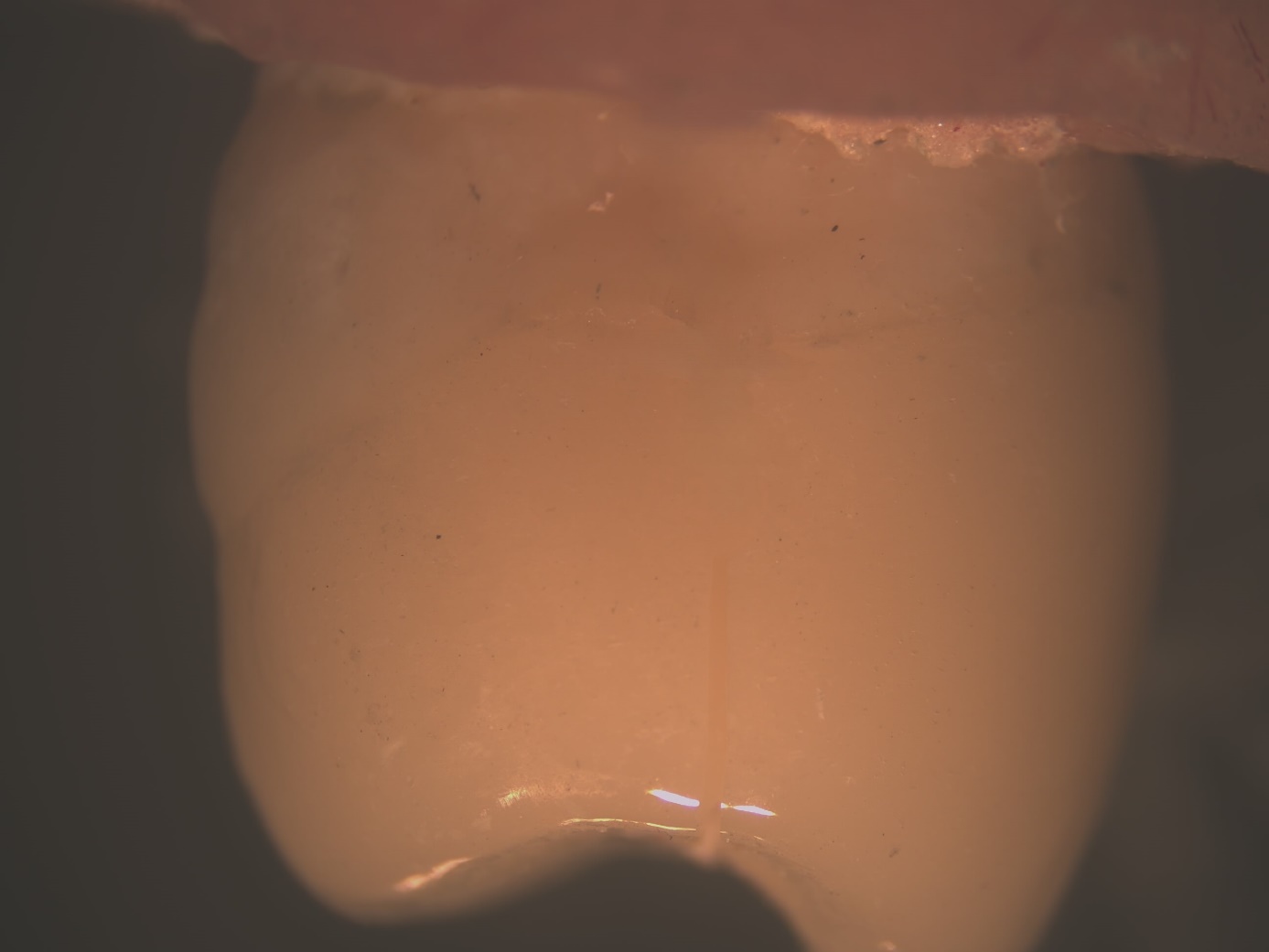


**Figure 8-A:** Full, uncropped image corresponding to the experimental setup. This figure shows the complete structure including all relevant anatomical or experimental details. No modifications were applied to the image beyond uniform contrast enhancement. All visible regions are retained to ensure reproducibility and transparency.


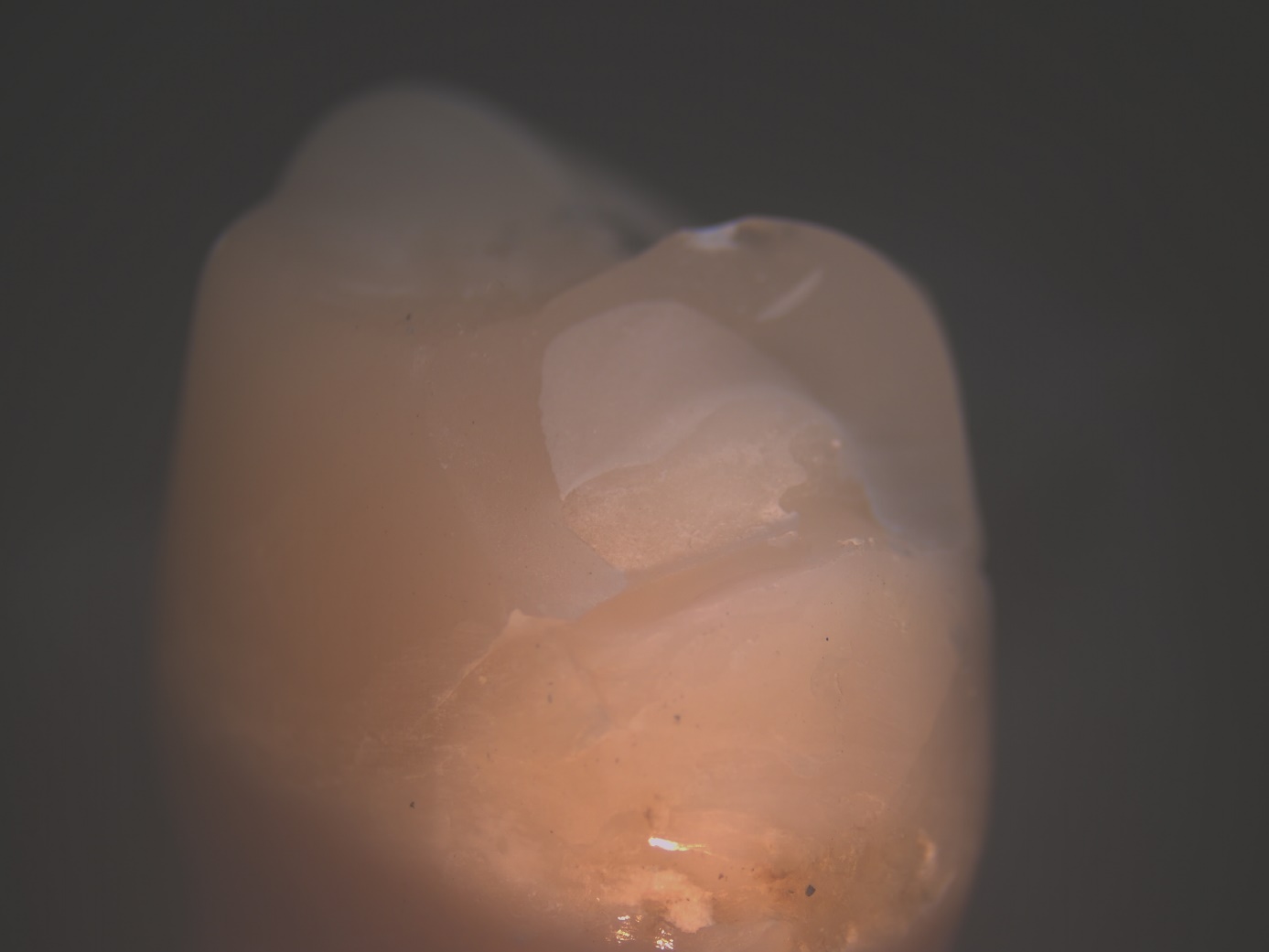


**Figure 8-B:** Full, uncropped image corresponding to the experimental setup. This figure shows the complete structure including all relevant anatomical or experimental details. No modifications were applied to the image beyond uniform contrast enhancement. All visible regions are retained to ensure reproducibility and transparency.


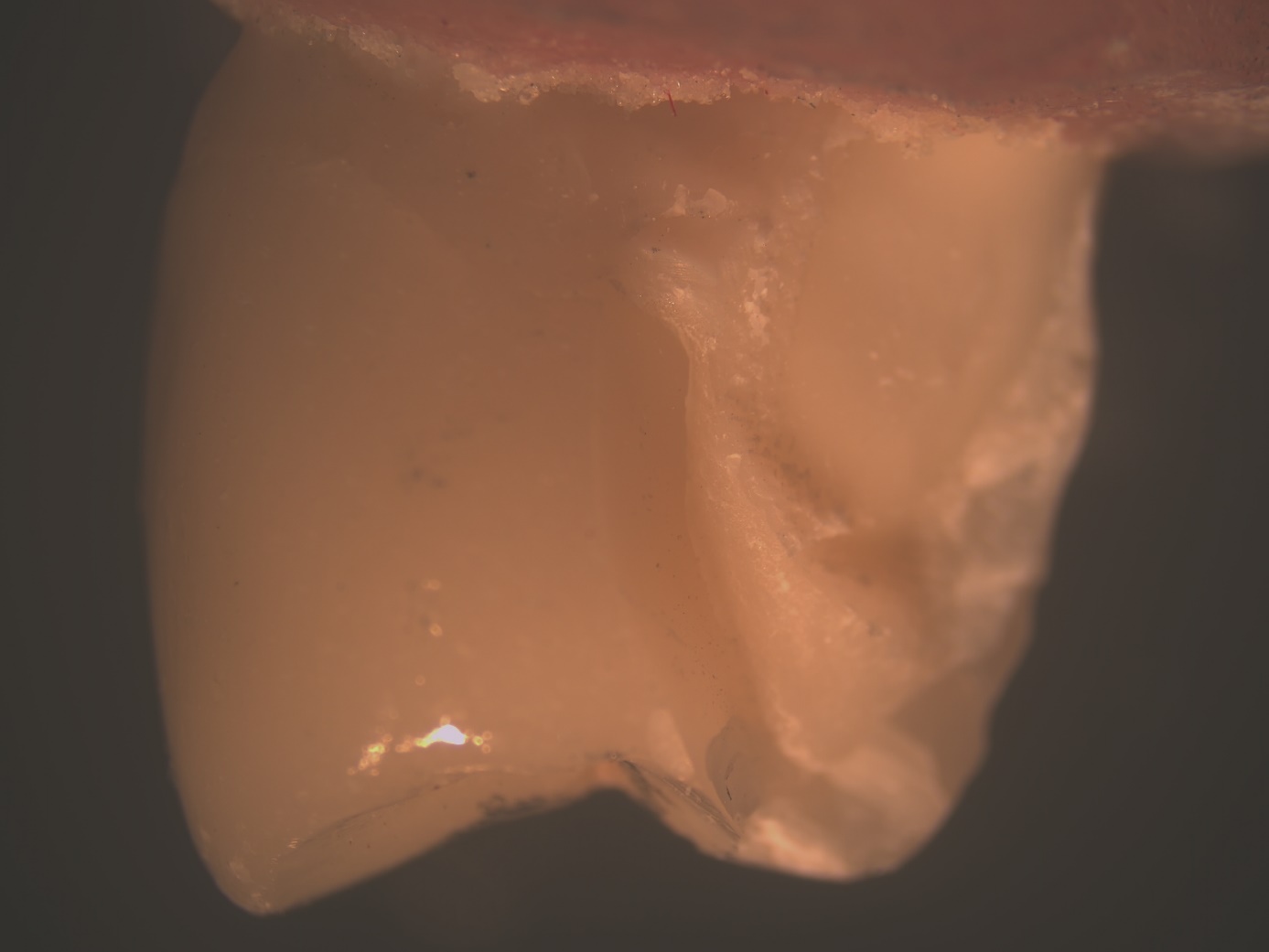


**Figure 8-C:** Full, uncropped image corresponding to the experimental setup. This figure shows the complete structure including all relevant anatomical or experimental details. No modifications were applied to the image beyond uniform contrast enhancement. All visible regions are retained to ensure reproducibility and transparency.


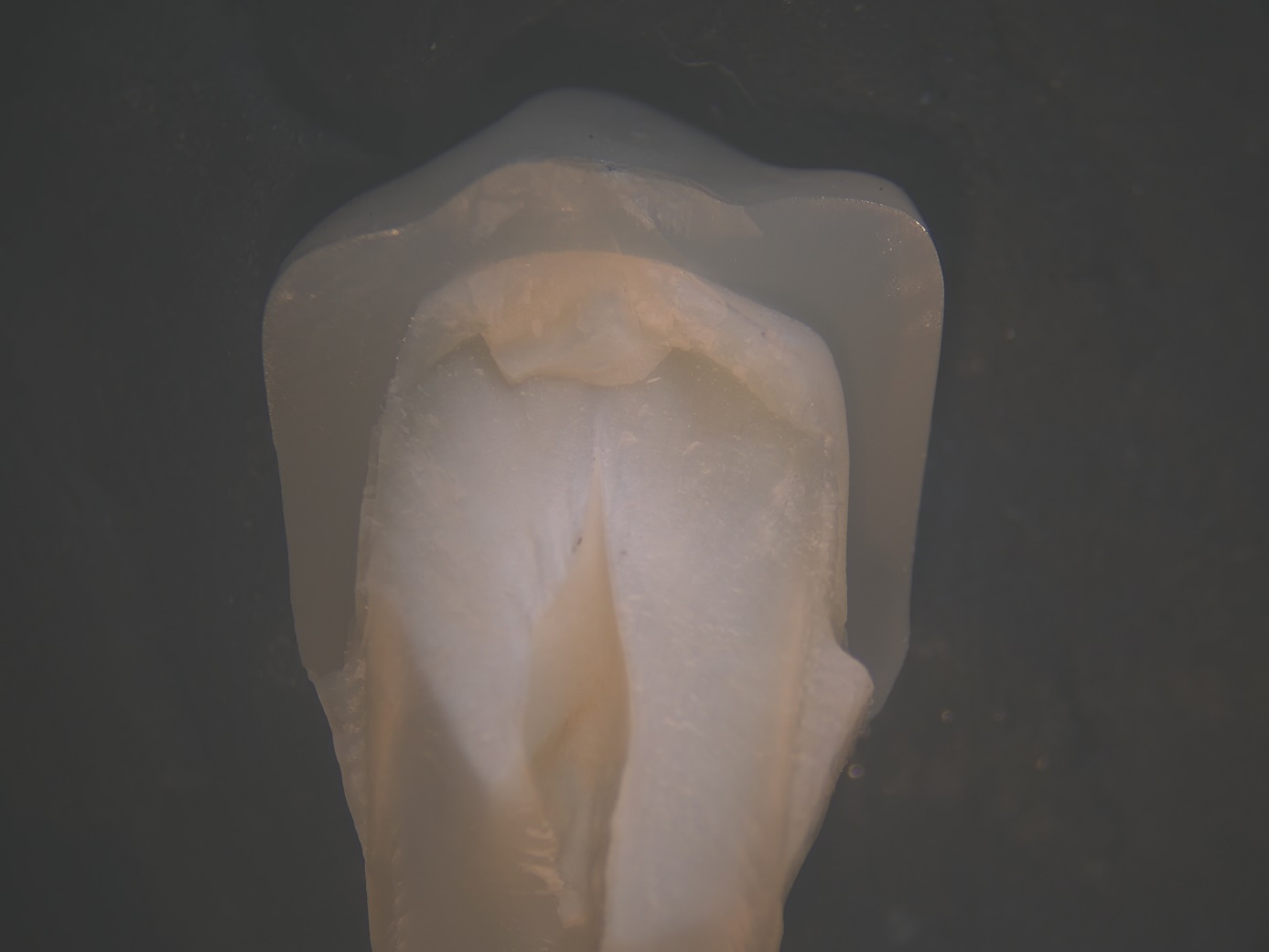


**Figure 8-D:** Full, uncropped image corresponding to the experimental setup. This figure shows the complete structure including all relevant anatomical or experimental details. No modifications were applied to the image beyond uniform contrast enhancement. All visible regions are retained to ensure reproducibility and transparency.


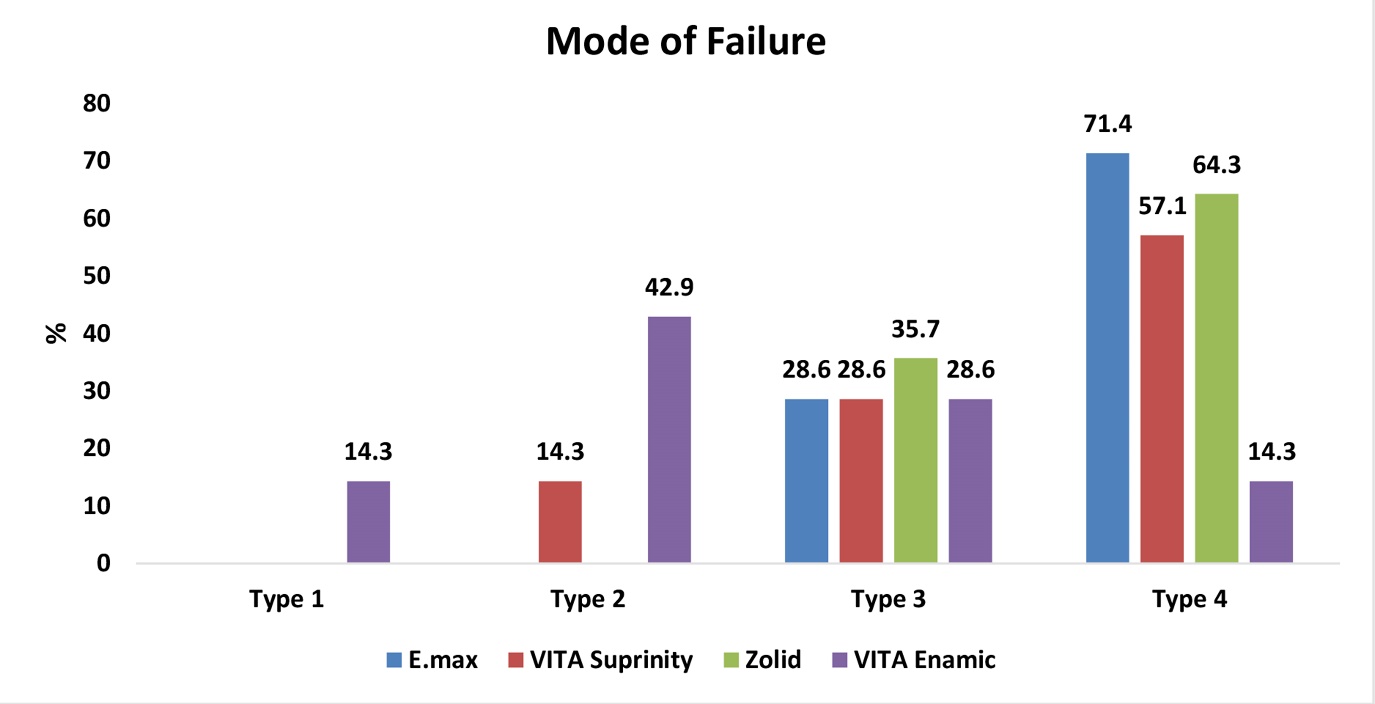


**Figure 9:** Chart showing a comparison of mode of failure between four studied groups

Illustrations showing, Unprocessed and uncropped original blot images are provided as Supplementary Data. Each blot corresponds to the figures presented in the main manuscript and includes all lanes, including those not shown in the final figure. Molecular weight markers are visible, and no selective adjustments were made. The data are presented in their original form to ensure transparency, reproducibility, and integrity of the experimental results.

**Vonlay preparation, scanning & restoration designing**

**Group I: IPS-EMAX CAD**

| **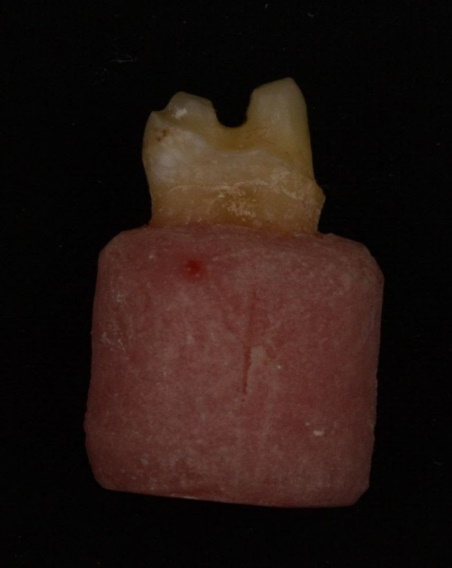** | **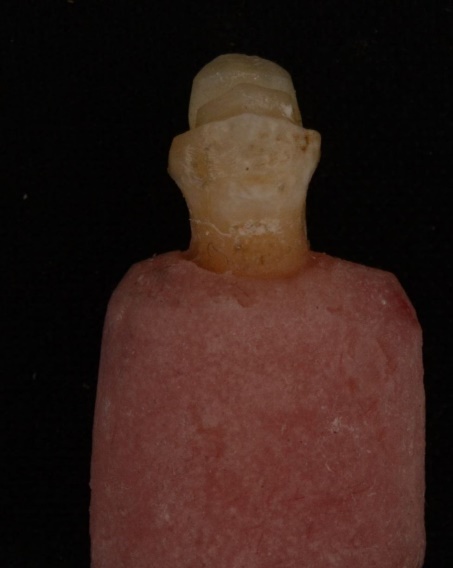** |
| --- | --- |
| Proximal view of the preparation. | Palatal view of the preparation. |
|  |  |
| **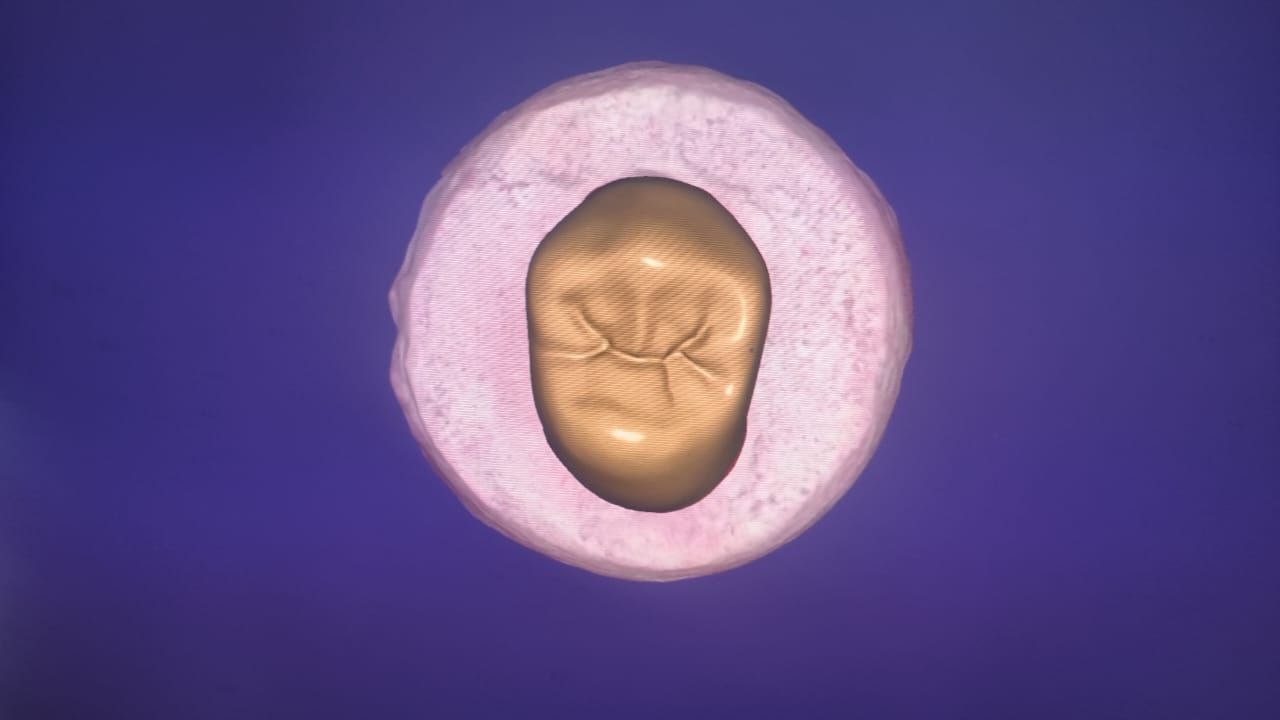** | **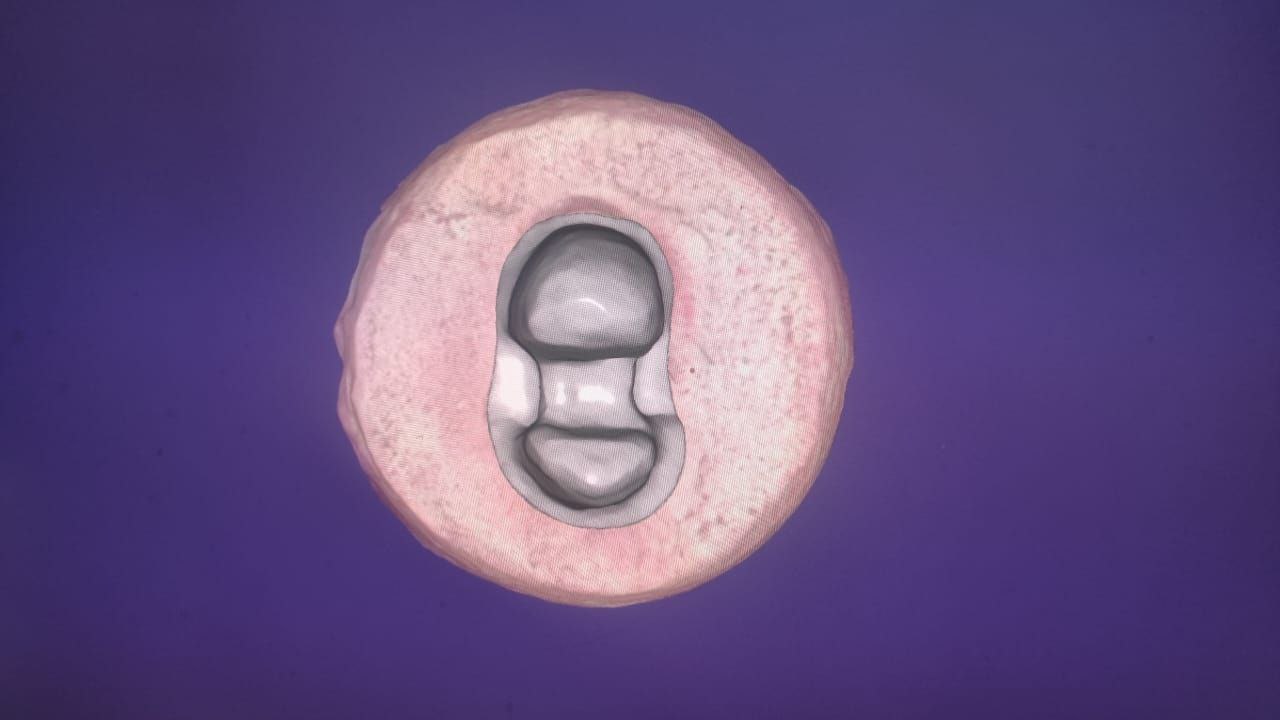** |
| Designing the restoration. | Scanning the preparation. |

**Vonlay preparation, scanning & restoration designing**

**Group II: Vita Suprinity**

| **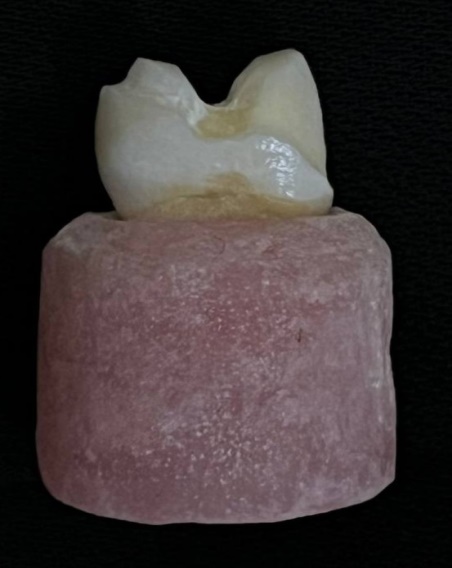** | **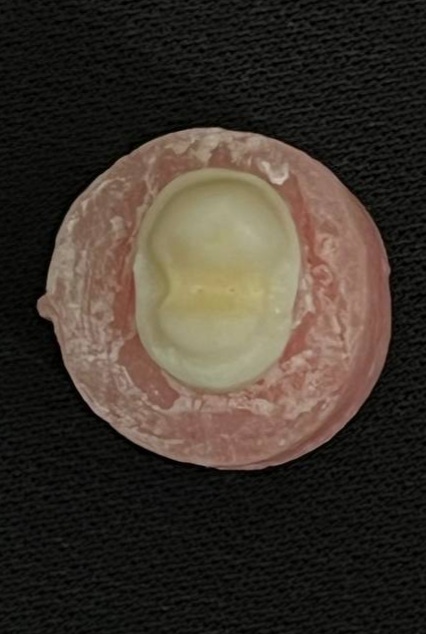** |
| --- | --- |
| Proximal view of the preparation. | Occlusal view of the preparation. |
|  |  |
| **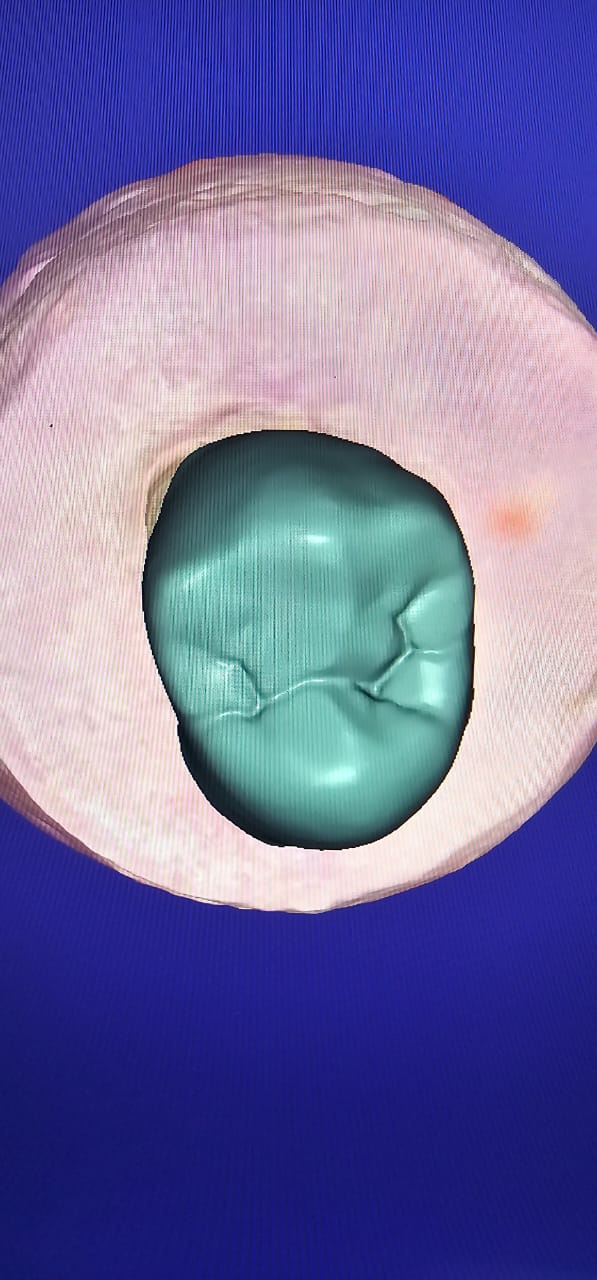** | **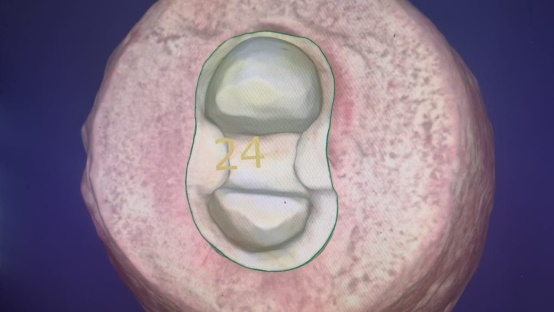** |
| Designing the restoration. | Scanning the preparation. |

**Vonlay preparation, scanning & restoration designing**

**Group III: ZOLID**

| **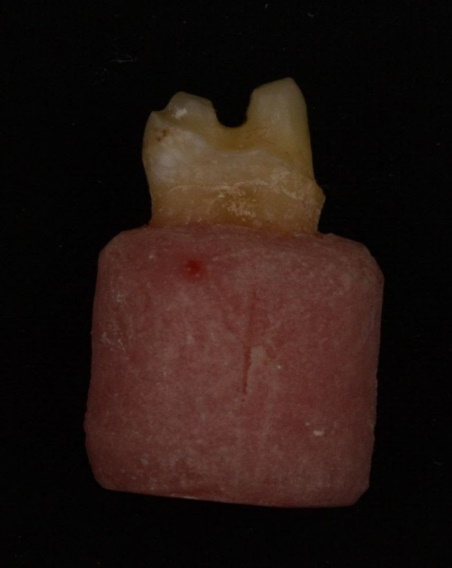** | **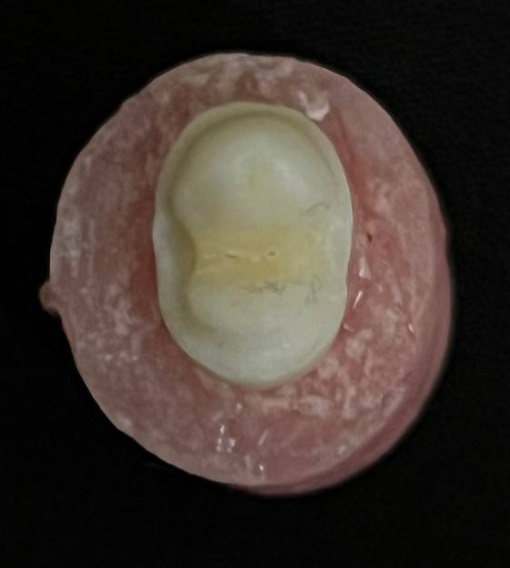** |
| --- | --- |
| Proximal view of the preparation. | Occlusal view of the preparation. |
|  |  |
| **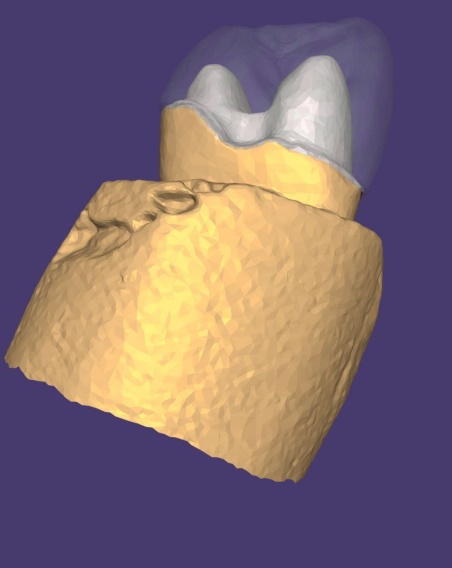** | **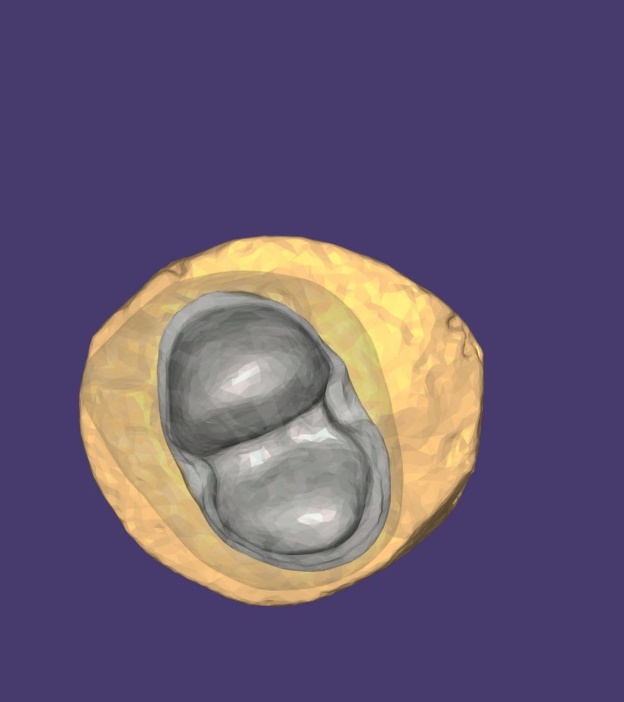** |
| Designing the restoration. | Scanning the preparation. |

**Vonlay preparation, scanning & restoration designing**

**Group IV: Vita Enamic**

| **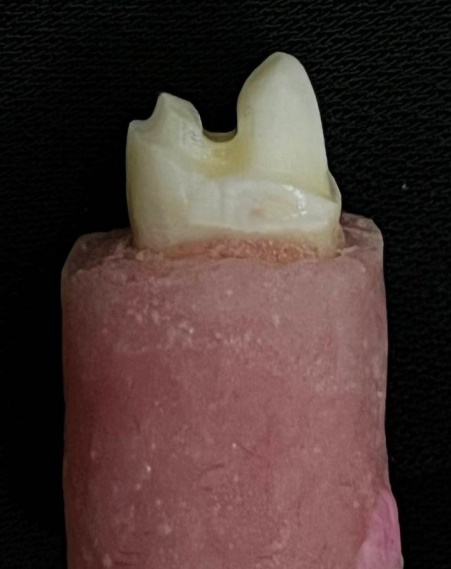** | **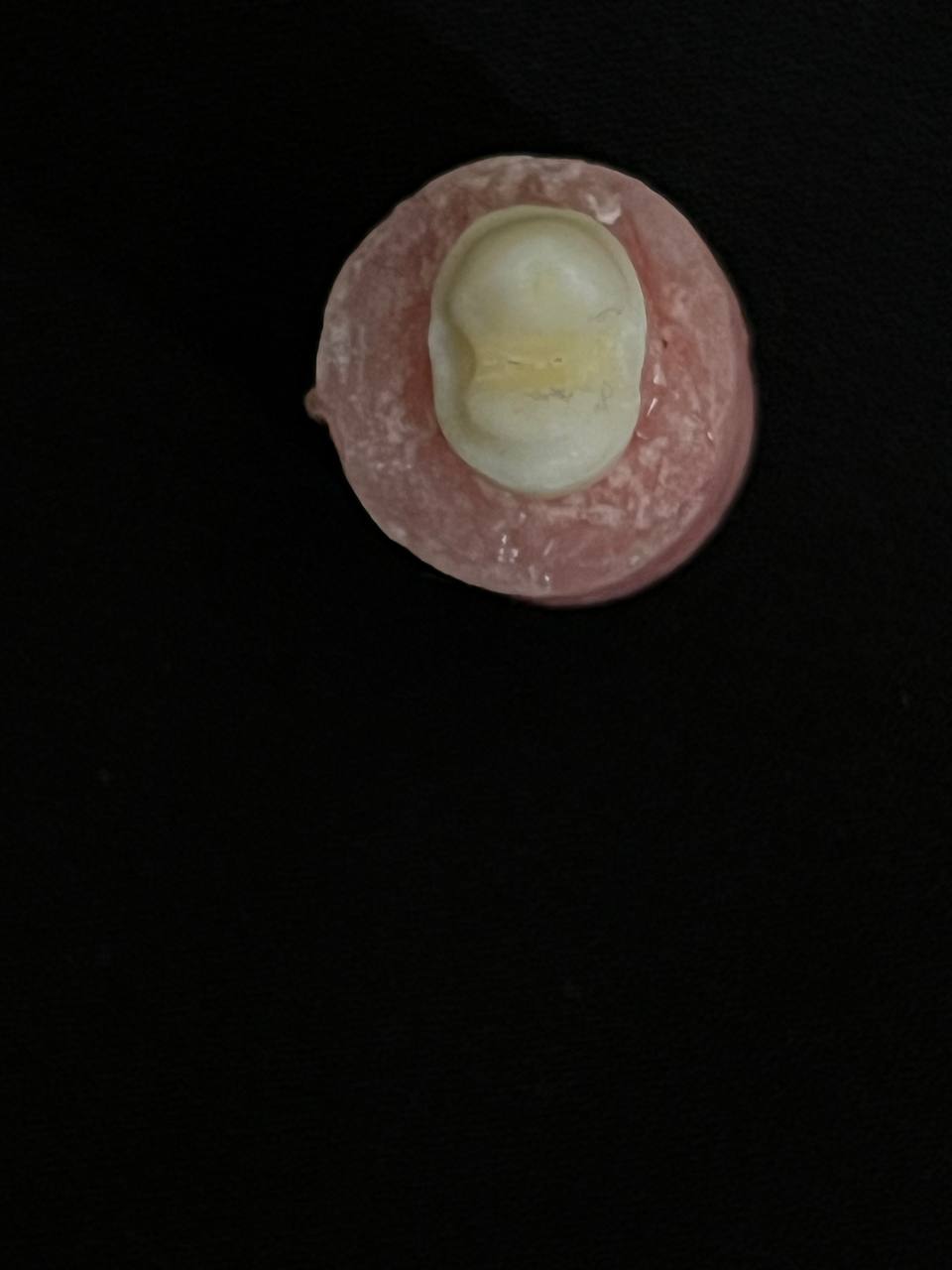** |
| --- | --- |
| Proximal view of the preparation. | Occlusal view of the preparation. |
|  |  |
| **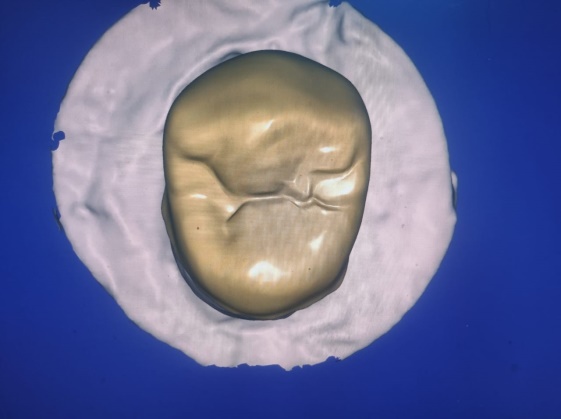** | **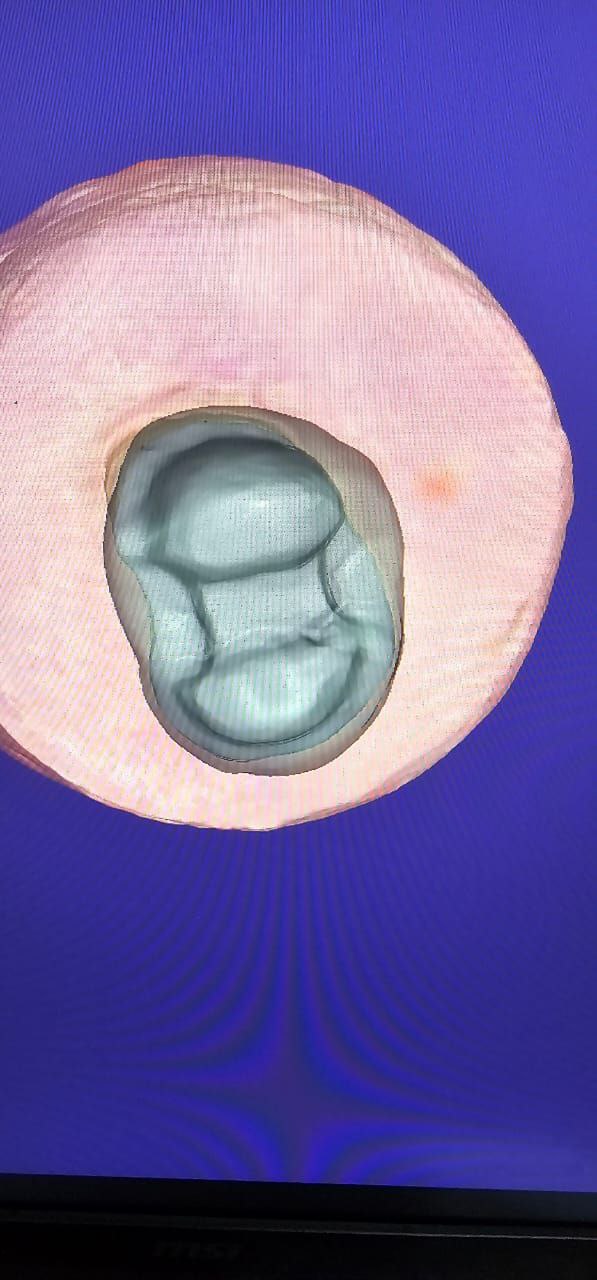** |
| Designing the restoration. | Scanning the preparation. |

**Vonlay restoration thickness checking**

| **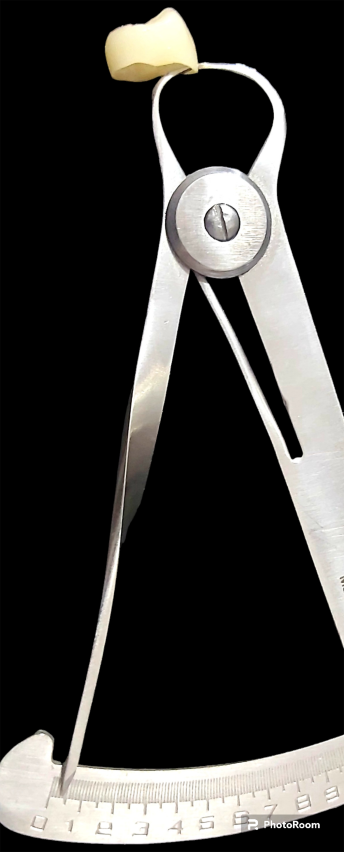** | **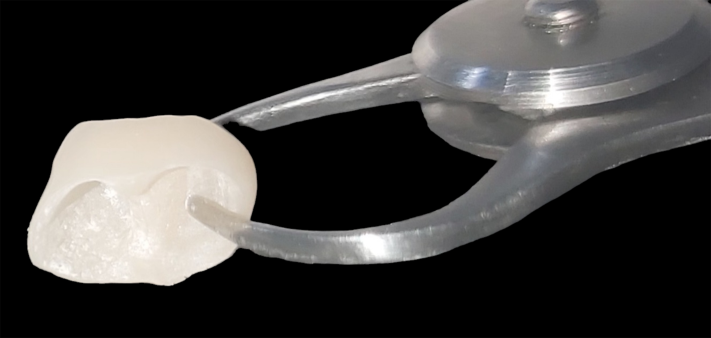** |
| --- | --- |
| Buccal aspect. | Fitting surface. |
|  |  |
| **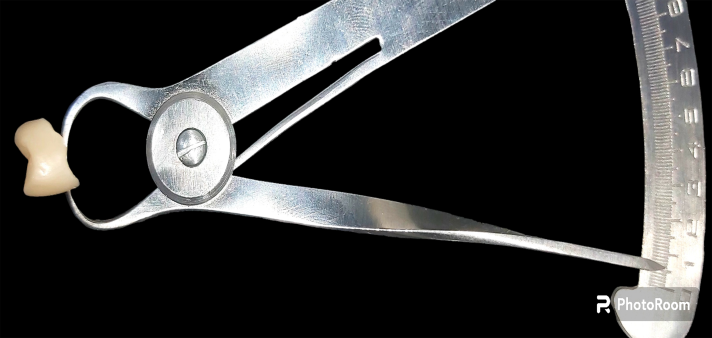** | **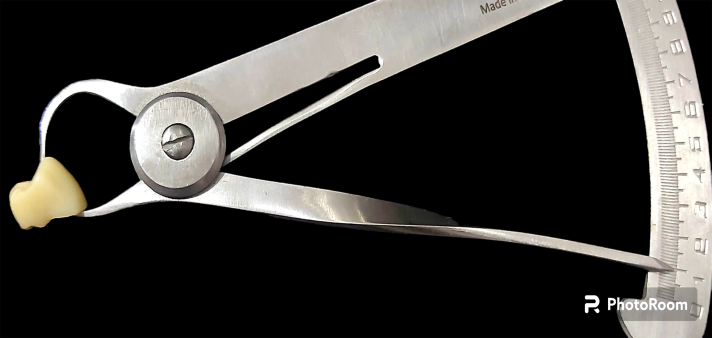** |
| Proximal view. | |

**
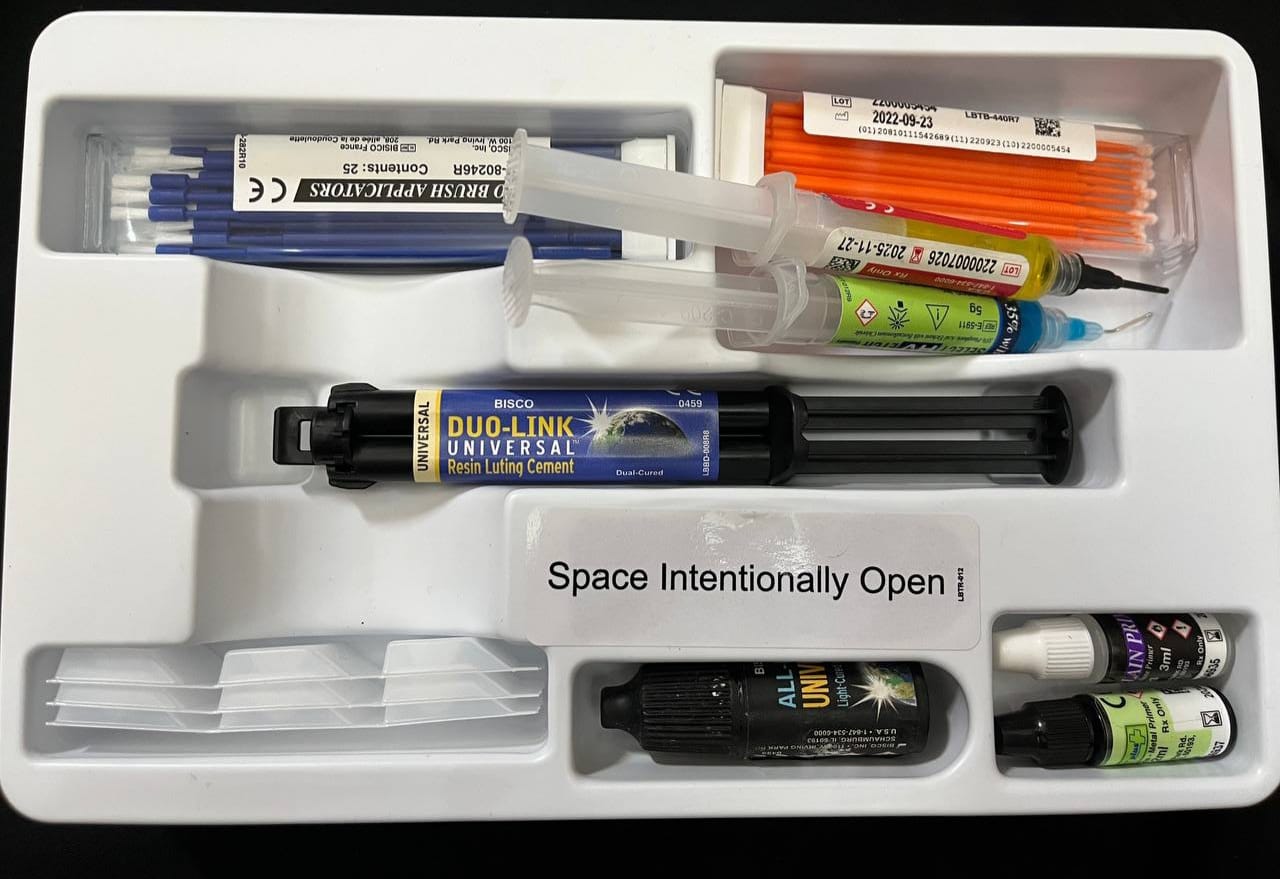
**

Adhesive resin cement: Duo-Link Universal, Universal Primer, Phosphoric Acid etchant 37%, Porcelain etchant, Porcelain Primer and Z prime Plus.

| **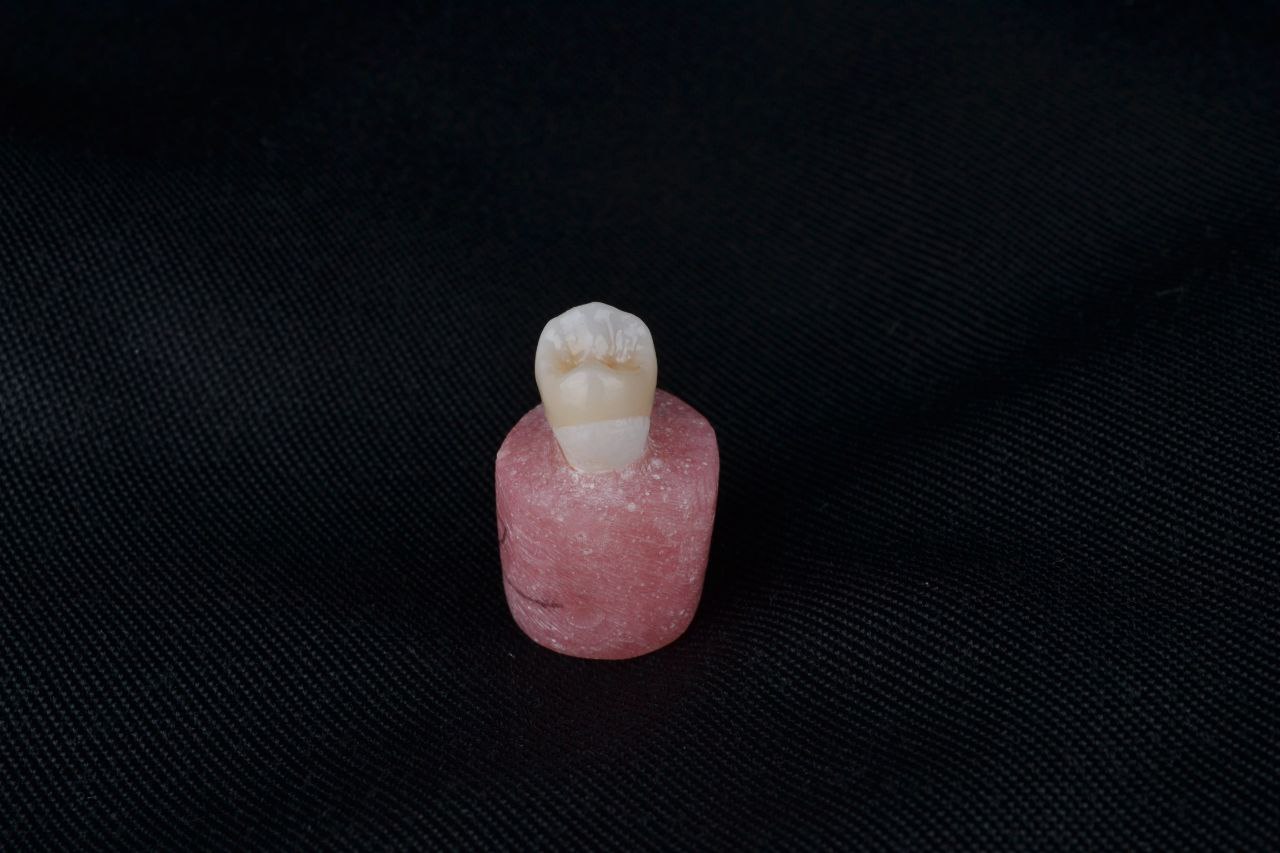** | **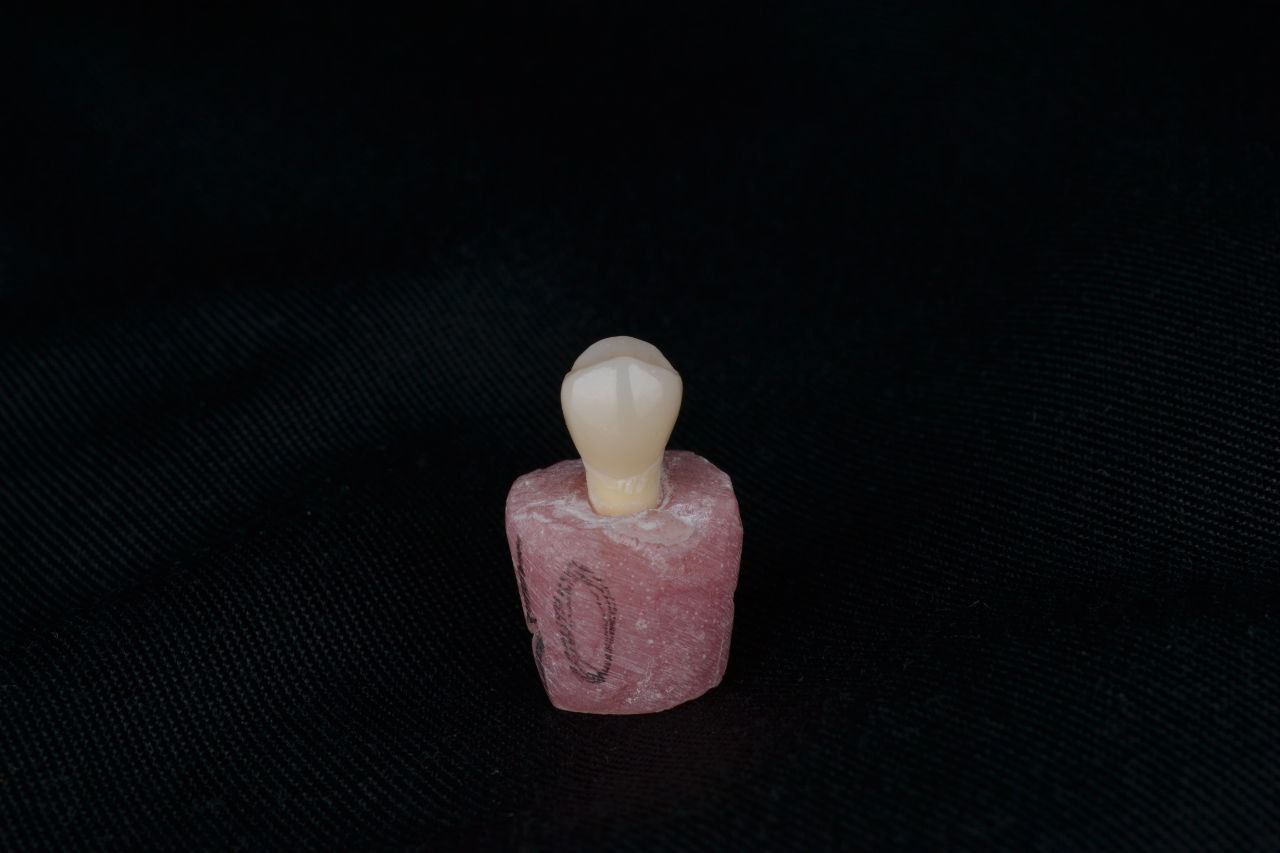** |
| --- | --- |
| Palatal view after cementation. | Buccal view after cementation. |
|  |  |
| **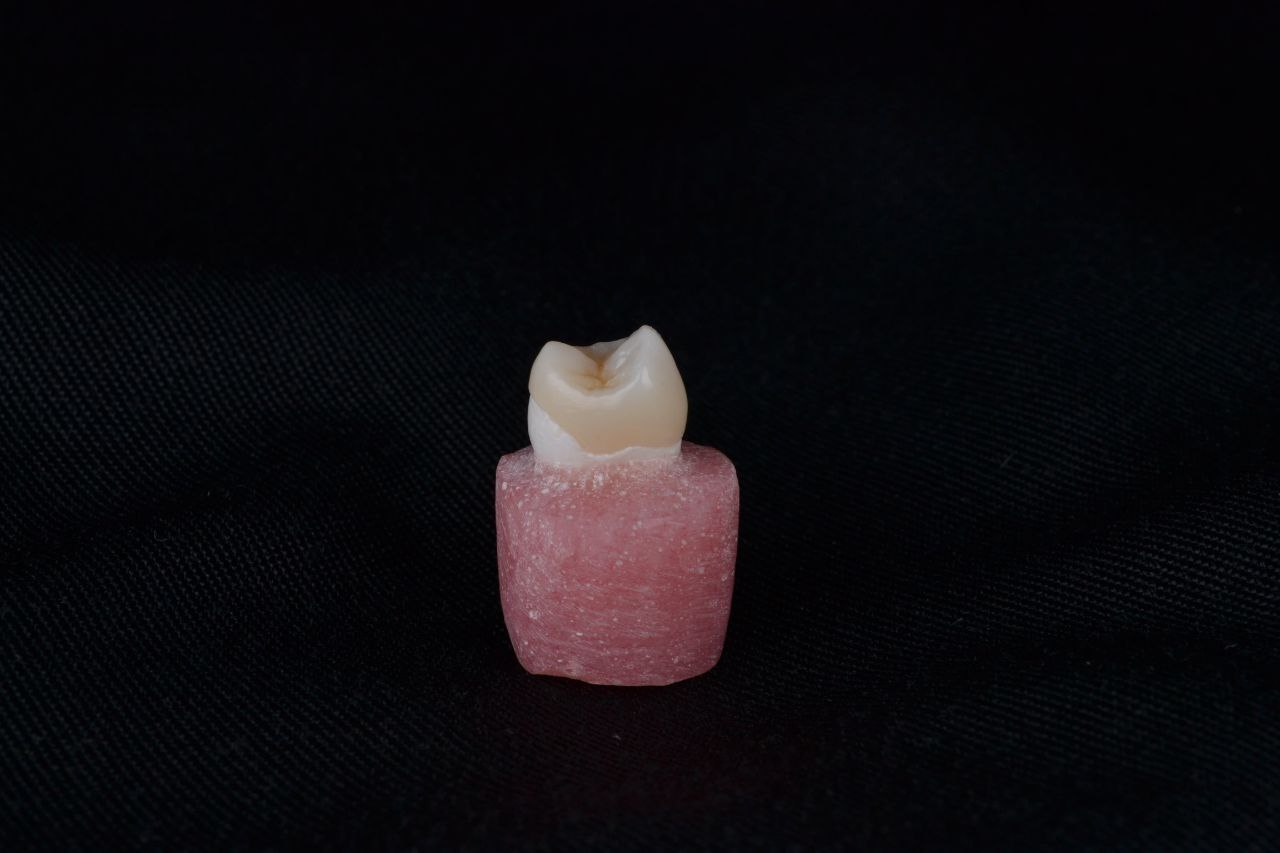** | **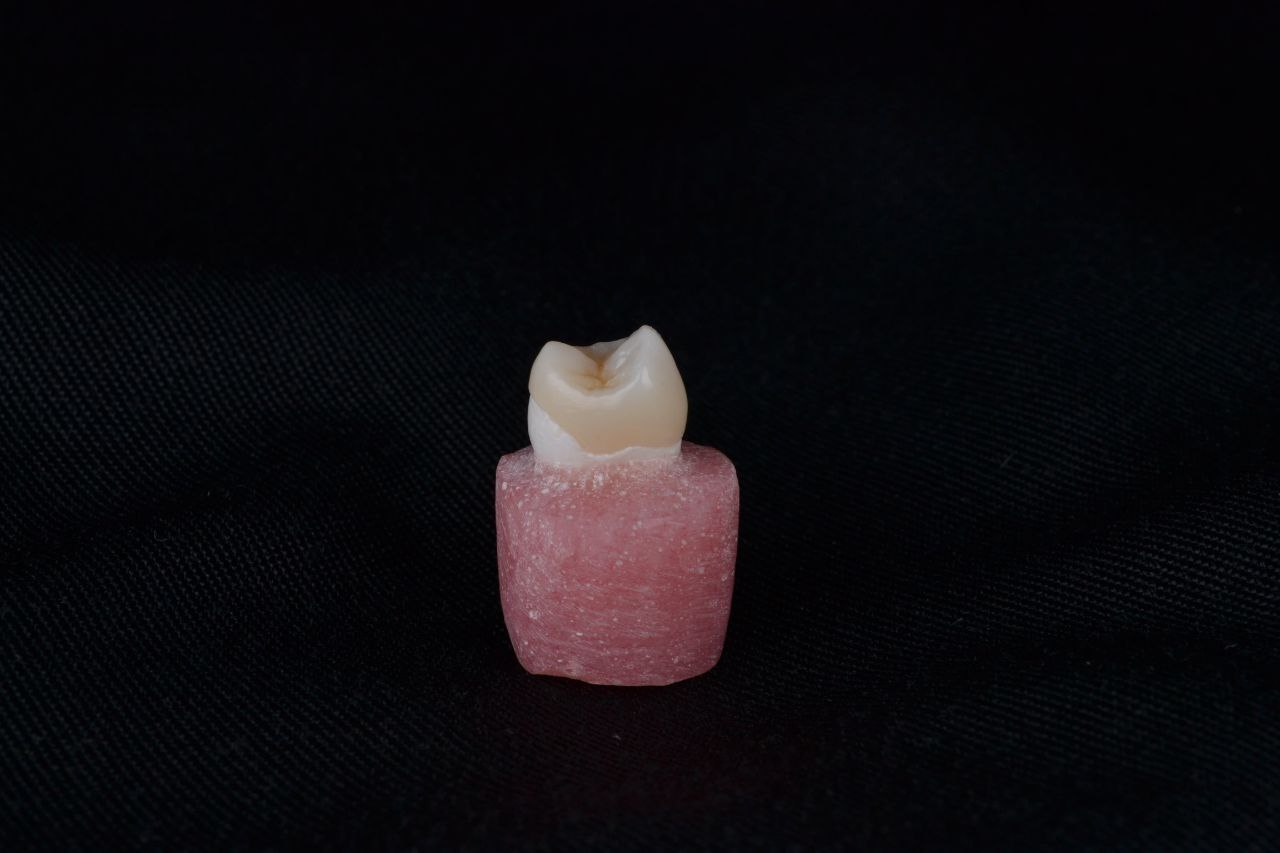** |
| Proximal (mesial) view after cementation. | Proximal (distal) view after cementation. |

| **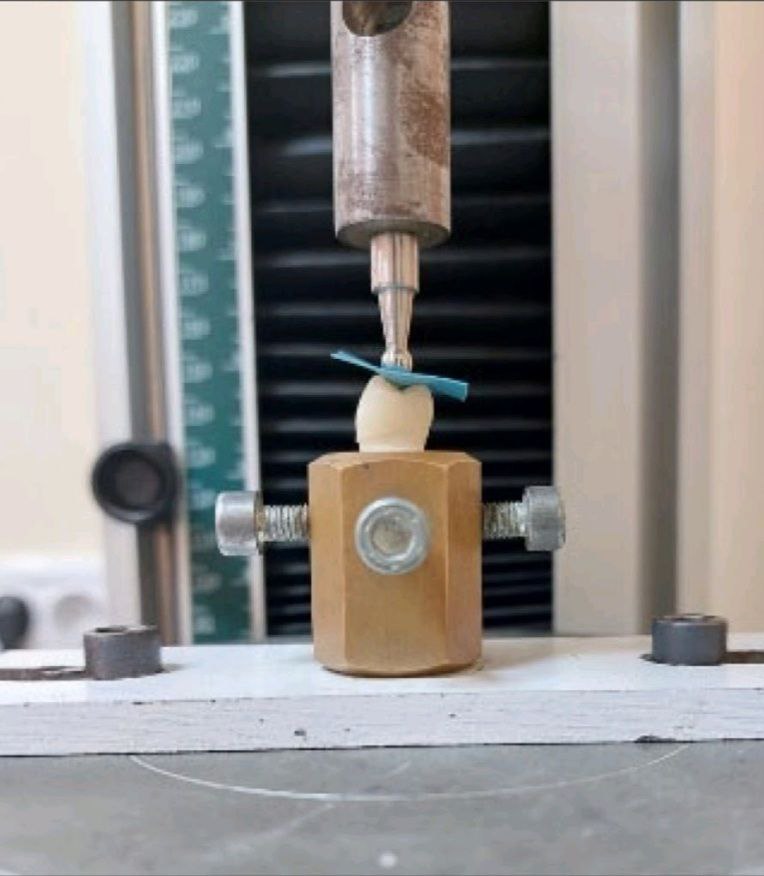** | **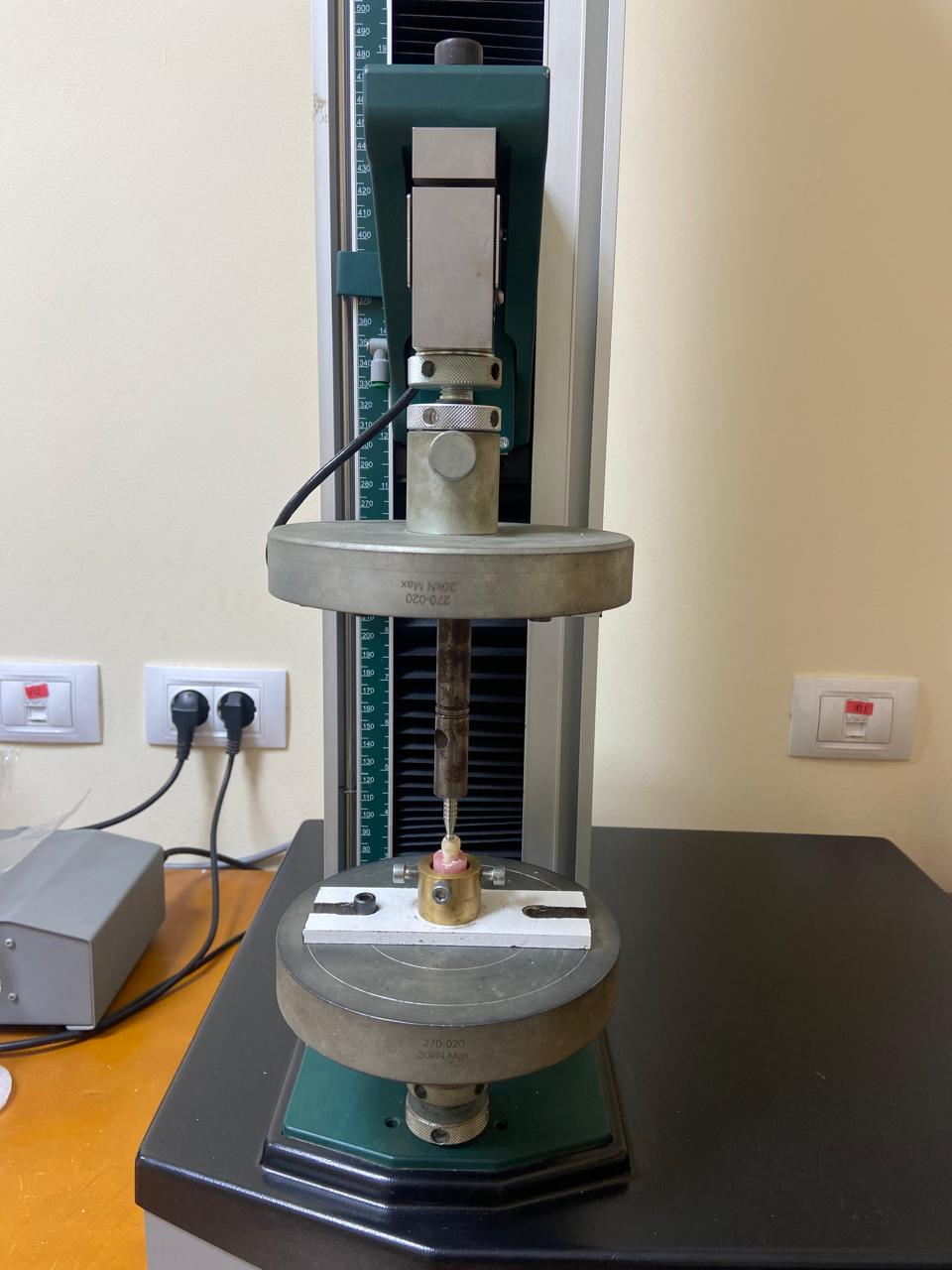** |
| --- | --- |
| Fracture resistance of Vonlay restoration. | Fracture resistance of Vonlay restoration. |
|  |  |
| **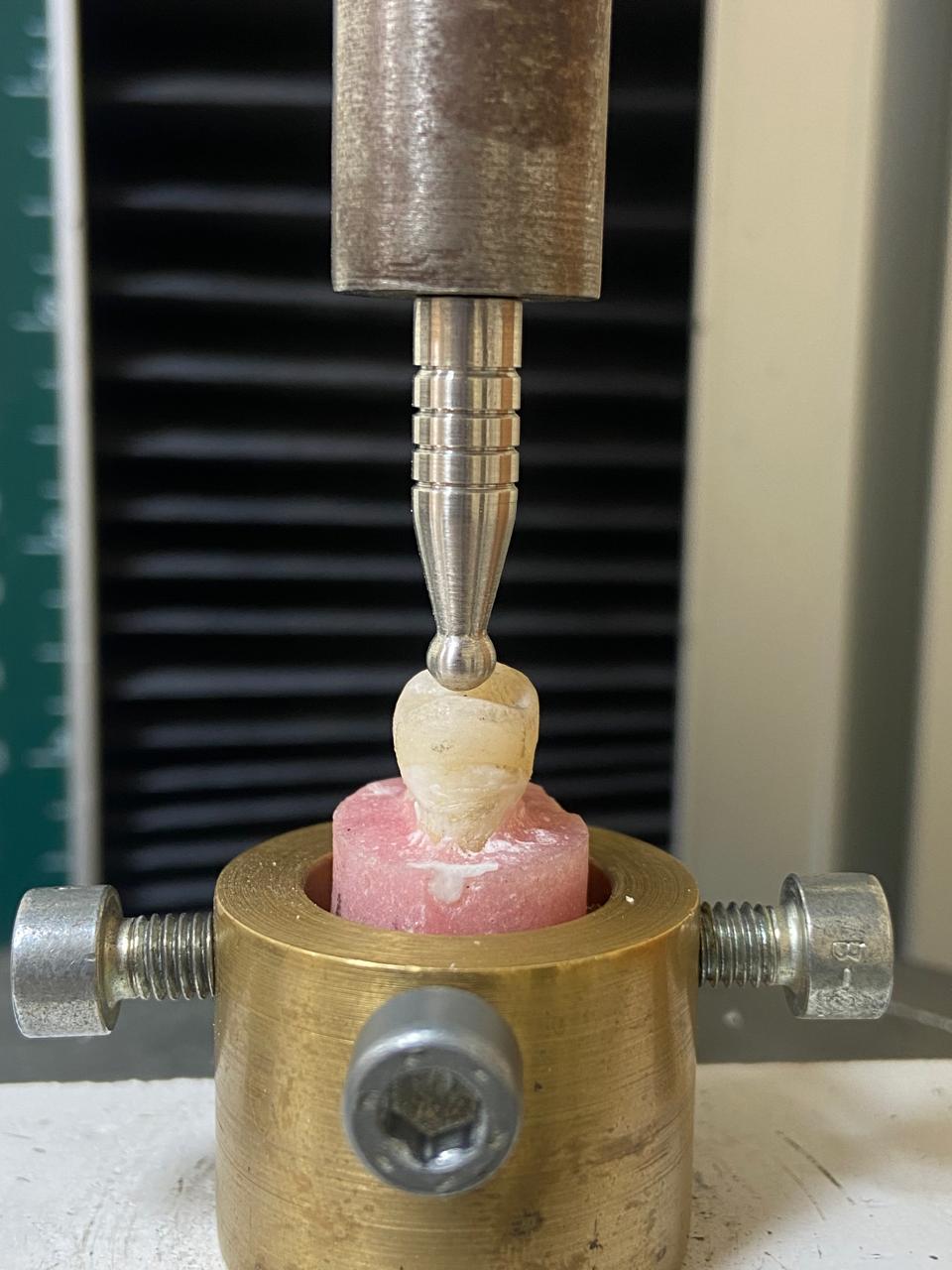** | **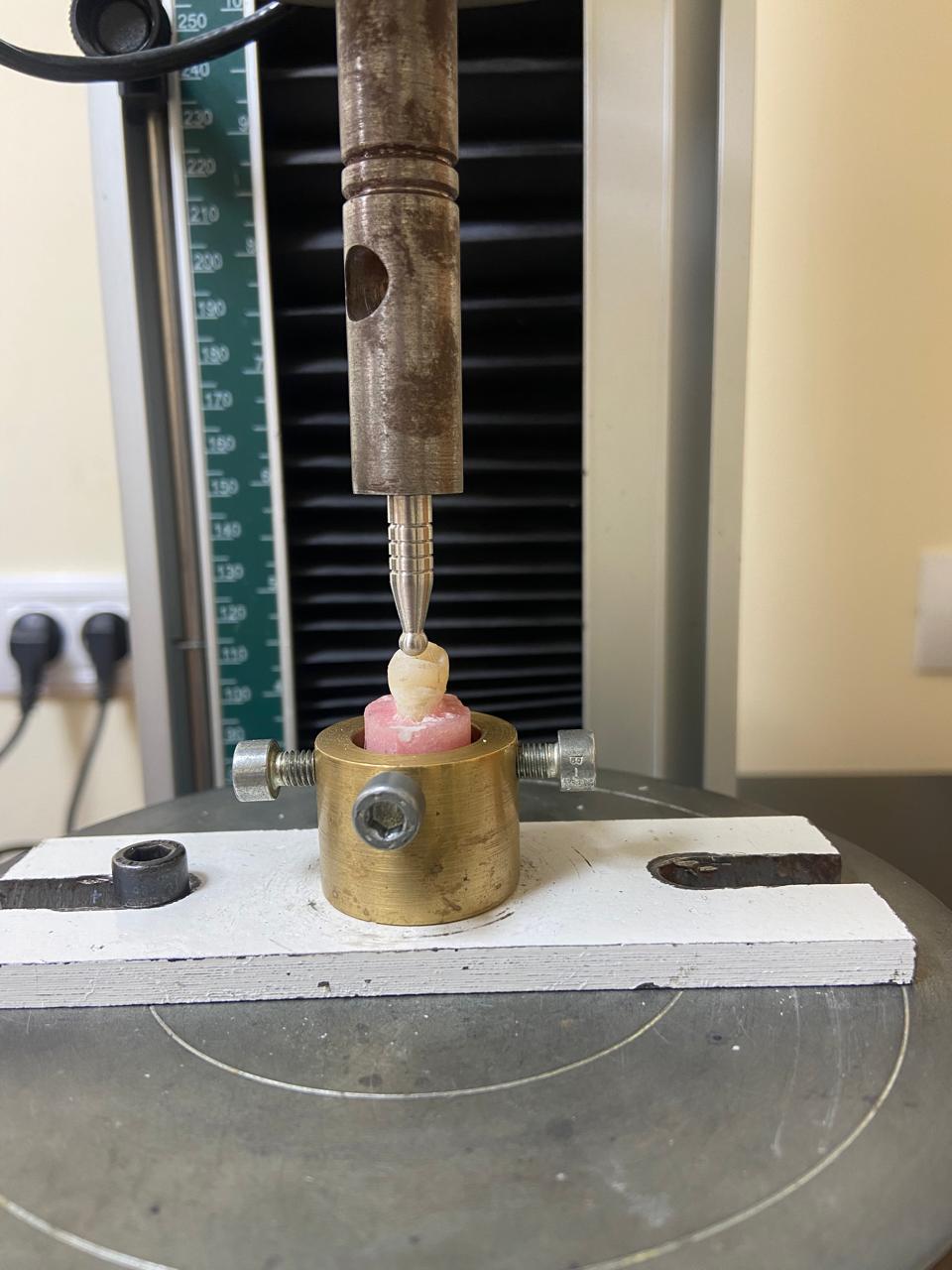** |
| Fracture resistance of Vonlay restoration. | Fracture resistance of Vonlay restoration. |

| **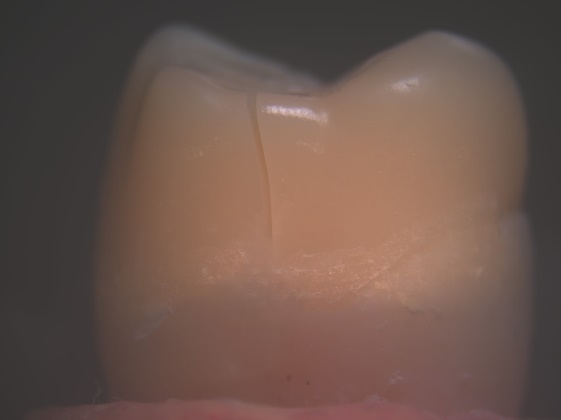** | **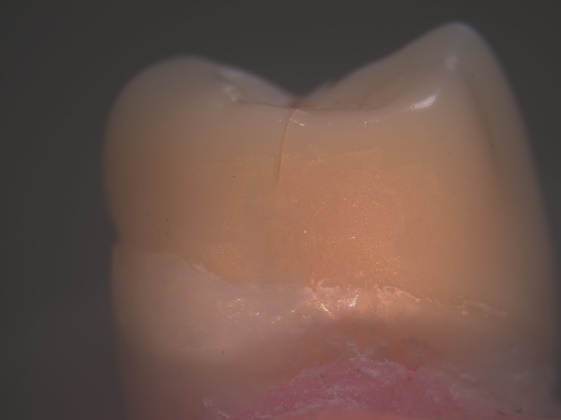** |
| --- | --- |
| **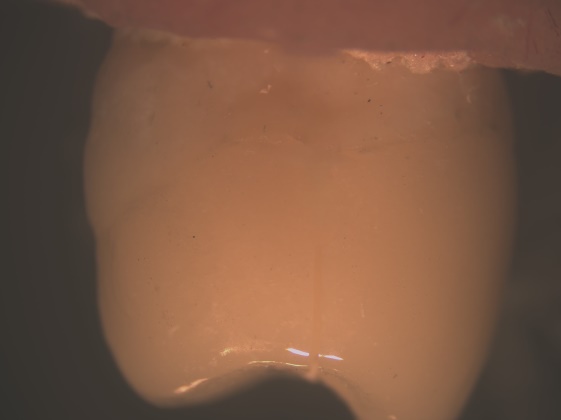** | **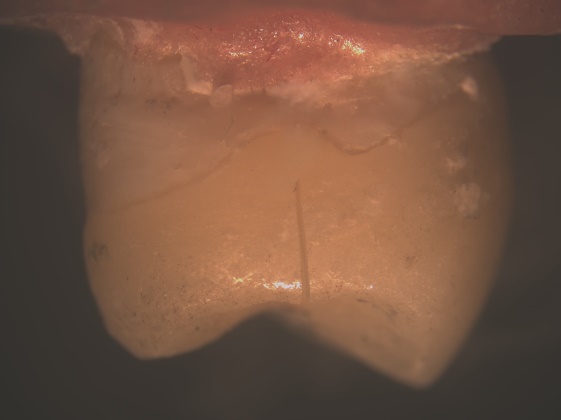** |

Failure mode of Mode I: Extensive crack formation within the ceramic by using Stereomicroscope 20x magnification.

| **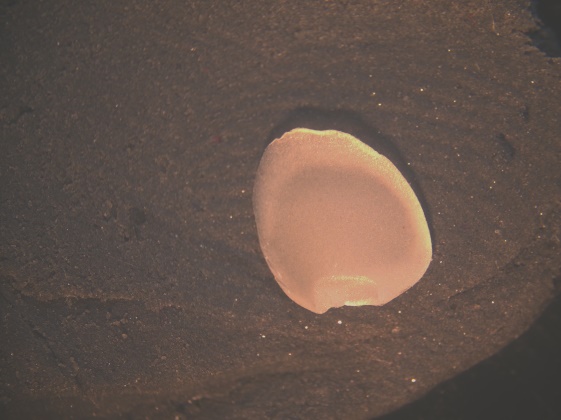** | **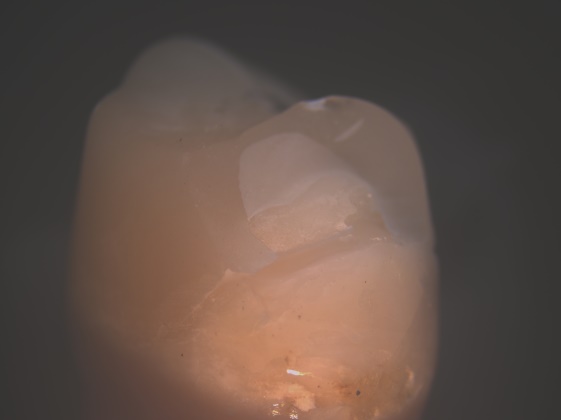** |
| --- | --- |
| **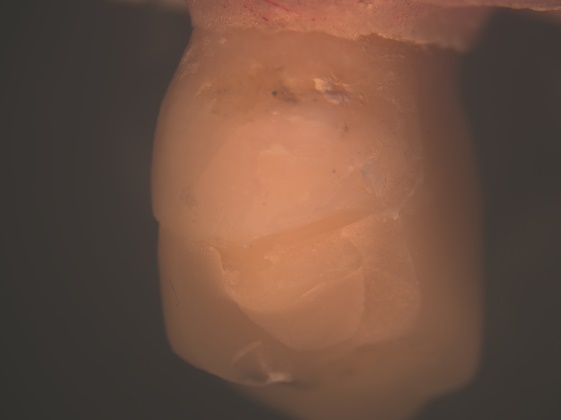** | **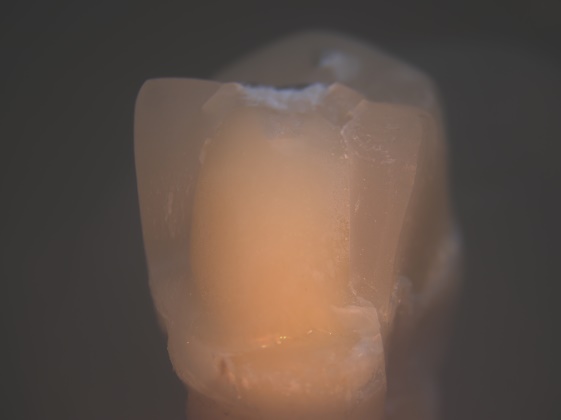** |

Failure mode of Mode II: Cohesive fracture within the ceramic by using Stereomicroscope 20x magnification.

| **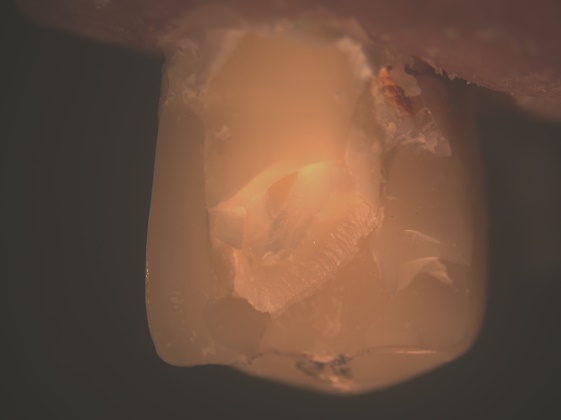** | **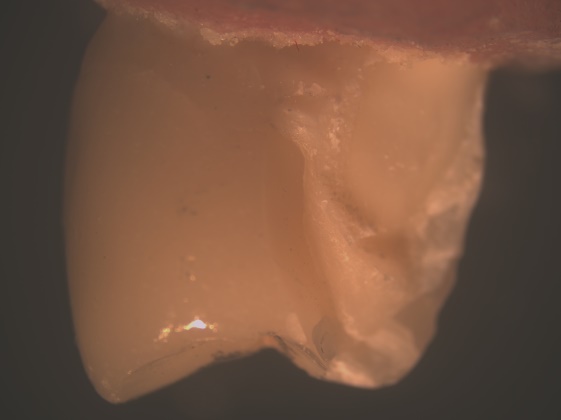** |
| --- | --- |
| **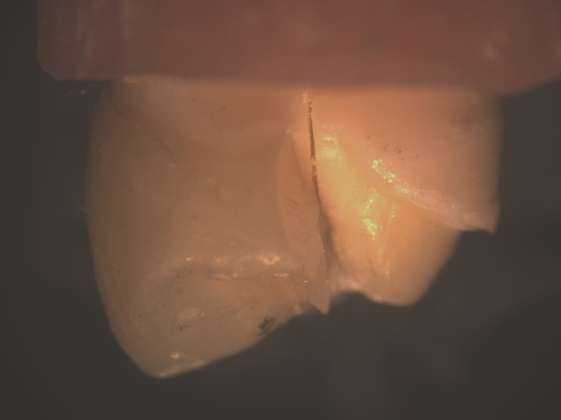** | **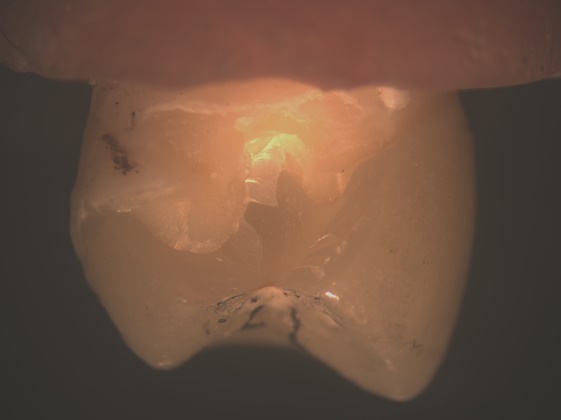** |

Failure mode of Mode III: Fracture within the ceramic and tooth structures by using Stereomicroscope 20x magnification.

| **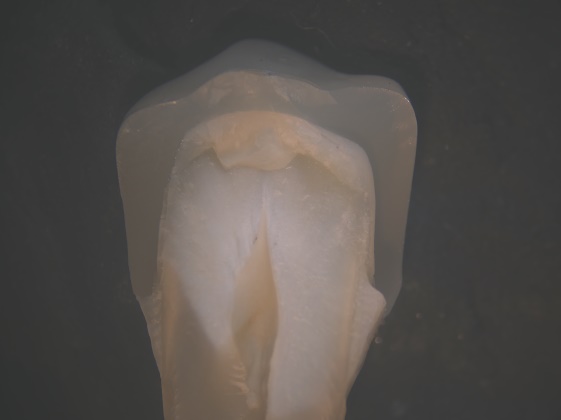** | **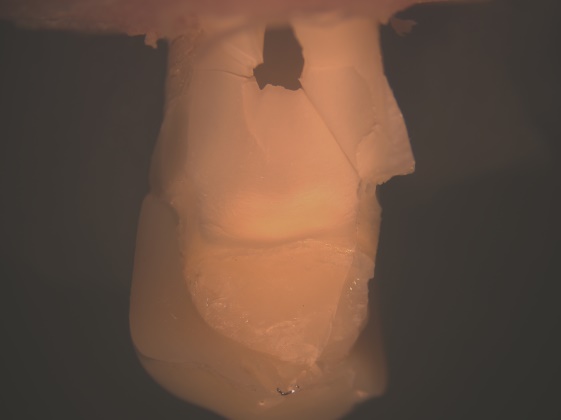** |
| --- | --- |
| **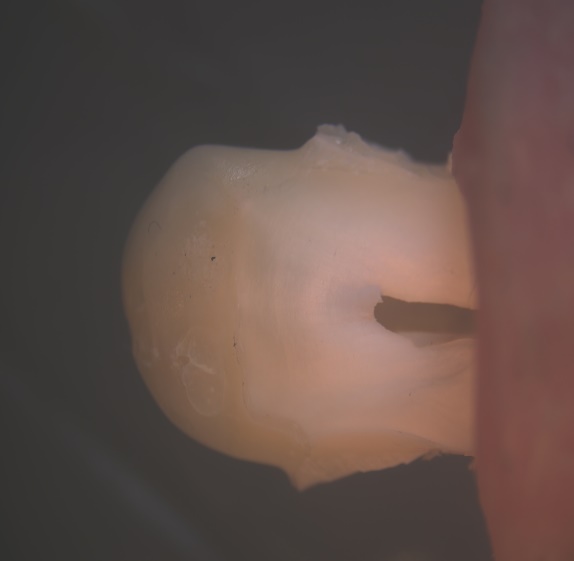** | **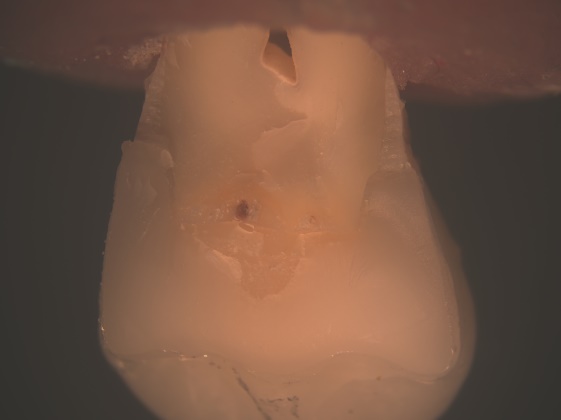** |

Failure mode of Mode IV: Longitudinal ceramic and tooth fracture involving the root by using Stereomicroscope 20x magnification.

**Failure mode taken by digital camera**

| 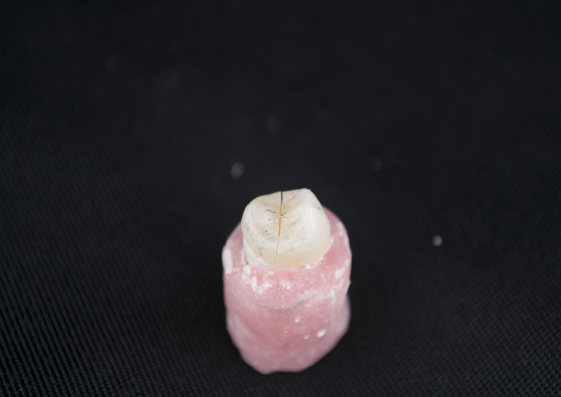 | 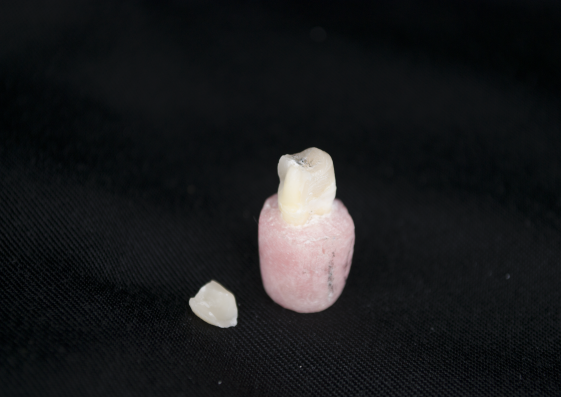 |
| --- | --- |
| Mode failure Type I | Mode failure Type II |
|  |  |
| 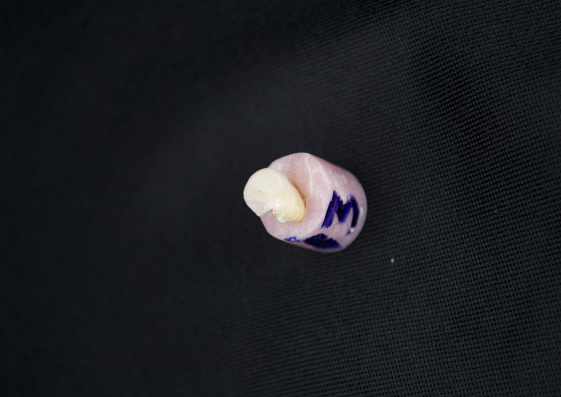 | 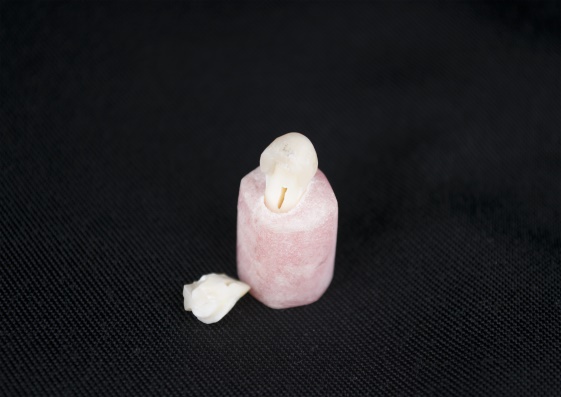 |
| Mode failure Type III | Mode failure Type IV |
